# New Hypervariable SSR Markers for Diversity Analysis, Hybrid Purity Testing and Trait Mapping in Pigeonpea [*Cajanus cajan* (L.) Millspaugh]

**DOI:** 10.3389/fpls.2017.00377

**Published:** 2017-03-31

**Authors:** Abhishek Bohra, Rintu Jha, Gaurav Pandey, Prakash G. Patil, Rachit K. Saxena, Indra P. Singh, D. Singh, R. K. Mishra, Ankita Mishra, F. Singh, Rajeev K. Varshney, N. P. Singh

**Affiliations:** ^1^ICAR-Indian Institute of Pulses Research (IIPR)Kanpur, India; ^2^International Crops Research Institute for the Semi-Arid TropicsHyderabad, India; ^3^ICAR-Indian Agricultural Statistics Research InstituteNew Delhi, India

**Keywords:** pigeonpea, SSR, diversity, hybrid, polymorphism, genome

## Abstract

Draft genome sequence in pigeonpea offers unprecedented opportunities for genomics assisted crop improvement *via* enabling access to genome-wide genetic markers. In the present study, 421 hypervariable simple sequence repeat (SSR) markers from the pigeonpea genome were screened on a panel of eight pigeonpea genotypes yielding marker validation and polymorphism percentages of 95.24 and 54.11%, respectively. The SSR marker assay uncovered a total of 570 alleles with three as an average number of alleles per marker. Similarly, the mean values for gene diversity and PIC were 0.44 and 0.37, respectively. The number of polymorphic markers ranged from 39 to 89 for different parental combinations. Further, 60 of these SSRs were assayed on 94 genotypes, and model based clustering using STRUCTURE resulted in the identification of the two subpopulations (*K* = 2). This remained in close agreement with the clustering patterns inferred from genetic distance (GD)-based approaches i.e., dendrogram, factorial and principal coordinate analysis (PCoA). The AMOVA accounted majority of the genetic variation within groups (89%) in comparison to the variation existing between the groups (11%). A subset of these markers was implicated for hybrid purity testing. We also demonstrated utility of these SSR markers in trait mapping through association and bi-parental linkage analyses. The general linear (GLM) and mixed linear (MLM) models both detected a single SSR marker (CcGM03681) with *R*^2^ = 16.4 as associated with the resistance to *Fusarium* wilt variant 2. Similarly, by using SSR data in a segregating backcross population, the corresponding restorer-of-fertility (*Rf*) locus was putatively mapped at 39 cM with the marker CcGM08896. However, The marker-trait associations (MTAs) detected here represent a very preliminary type and hence demand deeper investigations for conclusive evidence. Given their ability to reveal polymorphism in simple agarose gels, the hypervariable SSRs are valuable genomic resource for pigeonpea research community, particularly in South Asia and East Africa where pigeonpea is primarily grown.

## Introduction

Pigeonpea [*Cajanus cajan* (L.) Millspaugh] is an important grain-legume crop grown in 7.03 mha area with a total production of 4.89 mt from tropical and subtropical regions of the world (FAOSTAT, 2014). India is the largest producer of pigeonpea, contributing 67.3% to the global production, followed by Myanmar, Malawi, and Kenya (FAOSTAT, 2014). Owing to its inherent capacity to grow in low input and rainfed conditions coupled with its multiple uses as food, feed, forage and fuel wood, pigeonpea serves as a valued cash crop for small scale and marginal farmers (Sameer Kumar et al., 2016). A deep and extensive root system of pigeonpea contributes to improve structural and physical properties of the soil, a feature that could be harnessed to check soil erosion (Krauss, 1936). From the 32 species known under the sub-tribe *Cajaninae, C. cajan* is the only cultivated species (Van der Maesen, 1990; Odeny, 2007). The breeding efforts aimed at improving pigeonpea led to the development and release of more than 100 improved varieties during last 50 years in India (Singh I. et al., 2016). However, the genetic gains from conventional breeding remained limited over same period of time (Varshney et al., 2013). This implies toward an urgent need to strengthen pigeonpea breeding program with the modern tools to improve their efficacy.

Molecular markers are among the pre-requisites to accelerate the crop breeding program through genomics assisted breeding (GAB). A range of marker systems including random amplified polymorphic DNA (RAPD) (Ratnaparkhe et al., 1995), restricted fragment length polymorphism (RFLP) (Nadimpalli et al., 1993), amplified fragment length polymorphism (AFLP) (Panguluri et al., 2006) and diversity array technology (DArT) (Yang et al., 2006), intron spanning region (ISR) (Kudapa et al., 2012), simple sequence repeat (SSR) (Saxena et al., 2010a) and single nucleotide polymorphism (SNP) (Kassa et al., 2012; Saxena et al., 2012) have been reported in pigeonpea. Among various marker systems available, SSRs are still preferred due to its abundance in genome, multi-allelic and co-dominant nature and ease of assaying (Gupta and Varshney, 2000). The SSR has been employed extensively in pigeonpea to study genetic diversity, linkage mapping and QTL analysis. However, majority of these SSRs were resolved in capillary-based detection system (Bohra et al., 2011; Njung'e et al., 2016).

According to Temnykh et al. (2001) SSRs with ≥20 nucleotides and <20 nucleotides are referred to as Class I (hypervariable) and Class II, respectively. The significance of hyper-variable SSRs in pigeonpea has been established owing to their ease of scoring in simple agarose gel (Singh et al., 2012; Dutta et al., 2013). Recently, decoding of the whole genome sequence of Asha (ICPL 87119) by Varshney et al. (2012) has enabled access to more than 23,000 primer pairs for SSRs (CcGM series). The present study aims to validate a set of 421 SSRs of this CcGM series. We further demonstrated the usefulness of these SSRs through investigating the genetic diversity and population structure of cultivated pigeonpea. The potential of these SSR markers in hybridity testing was also assessed on agarose gel. Also, we developed a partial linkage map of a backcross population using these SSRs and the *Rf* locus possibly responsible for A_2_-cytoplasmic male sterility (CMS) restoration was mapped. In parallel, these SSRs were also employed in association mapping to detect putative marker trait association (MTA) for *Fusarium* (*Fusarium udum*) wilt (FW) resistance in pigeonpea.

## Materials and methods

### Plant material

The experimental material comprised of 94 pigeonpea genotypes, which included 59 varieties, released during 1975–2015, 23 breeding lines/accessions, four exotic lines, four landraces, three fertility restorers, and two CMS lines (Table 1). These genotypes are relevant to pigeonpea breeding objectives owing to their suitability to different agro-climatic zones and having several traits of interests such as high yield and disease resistance etc (Supplementary Table 1). A subset of these genotypes (40) was screened against *Fusarium* wilt (FW) variant 2 during 2014–15 in the wilt sick field at IIPR, Kanpur (Supplementary Table 2). The 40 genotypes were selected because of their breeding value (extensively used by the breeders) for the improvement of resistance to FW. Few genotypes, e.g., IPA 8F, IPA 9F, and IPA 16F were registered with the National Bureau of Plant Genetic Resources (NBPGR), New Delhi as donors for FW resistance (Singh et al., 2011). Similarly, the genotypes like BDN 2, C11, MAL 13, and ICP 8863 are being used as differential set to characterize virulence of the wilt pathogen. The experiment was conducted in randomized block design (RBD) with two replications. The FW incidence was recorded on plants from November to February as the weather conditions during this period favor wilt incidence. Percent incidence was calculated using the formula:

$$\begin{array}{l} \%\text{ Disease incidence}=(\text{Number of infected plants/Total}\\ \text{ number of plants screened})\times \text{100} \end{array}$$

Arcsine transformation was used to normalize the data.

**Table 1:** **Pigeonpea genotypes used to investigate genetic diversity and population structure**.

**Type**	**Genotype names**	**Total number**
**Cultivars**		59
1975-1985	Prabhat, C 11, BDN 2, UPAS 120, GS 1, PT 221, TAT 10, LRG 30, AL 15, AL 201, TV 1, Manak, Pusa 84, CO 5, T 15-15	
1986-1995	ICP 8863, ICPL 87, Bahar, Pusa 33, Type 7, Abhaya (ICPL 332), Jagriti (ICPL 151), TTB 7, GT 1, JA 4, CO 6, GT 100, Pusa 855, Vamban 1, Sharad (DA 11), Pusa 9, Durga (ICPL 84031)	
1996-2005	JKM 7, Amar, NDA 1, Laxmi (ICPL 85063), Paras, Azad, MA 3, TS 3, Pusa 992, WRP 1, LRG 38, GT 101, MA 6, WRG 27, CORG 9701, BDN 708, NDA 2, MAL 13	
2006-2015	Pusa 2001, JKM 189, Vipula, PAU 881, VLArhar 1 (ICPL 88039), Pusa 2002-2, BRG 2, WRG 53, IPA 203	
Landraces	Allahabad Local, Banda Palera, JBP 13, Kudarat	4
Exotic lines	ICP 7124 (EC 109873), ICP 7148 (EC 109897), MN 5, MN 8	4
Restorers	AK 250173R, AK 250189R, AK 261354R	3
CMS lines	CORG 99052A, ICPA 2089A	2
Breeding lines/donors	AK 101, AK 22, AKP 1, D 20, DSLR 129, ICP 7035,	22
	ICPL 84023, ICPL 87154, IPA 16F, IPA 2012-01, KPL 43, PI 397430, Dholi Dwarf, ICP 89049, ICPL 91045, KPL 44, ICPL 88034, ICPL 11255, ICPL 20340, IPA 15F, IPA 8F, IPA 9F	

### Genomic DNA extraction

Genomic DNA was extracted from 94 pigeonpea genotypes for diversity analysis. Initially eight genotypes (Type 7, ICP 8863, PA 163A, ICPA 2089, AK 261322R, AK 261354R, AK 250189R, AK 250173R) were selected to assess the amplification status of SSR markers. For hybrid purity testing, DNA was extracted from 10 individuals each of three CMS hybrids *viz*. IPAH 16-06, IPAH 16-07, and IPH 15-03. To perform genetic linkage analysis, DNA from 102 individuals of a backcross population [ICPL 88039A × (ICPL 88039A × AK 250189R)] was also extracted. Also, to gain insights about within genotype variability in landraces DNA was isolated from 15 individuals each of six genotypes (two varieties and four landraces). Genomic DNA was isolated from young leaves according to Cuc et al. (2008). The quantity and quality of DNA was estimated through electrophoresis using 0.8% agarose gel (Sigma-Aldrich, St. Louis, USA).

### SSR analysis

For amplification of genomic DNA, a reaction mixture of 10 μl volume was prepared using 5.9 μl of sterilized distilled water, 1.0 μl template DNA (25 ng), 0.5 μl of forward and 0.5 μl of reverse primer (5 μM), 1.0 μl 10 × PCR buffer (10 mM Tris-HCl, 50 mM KCl, pH 8.3), 1.00 μl dNTP mix (0.2 mM each of dATP, dGTP, dCTP, and dTTP), and 0.1 μl *Taq* polymerase (5 U/μl) (Thermo Scientific, Mumbai, India). The amplification was carried out in G-40402 thermo cycler (G-STORM, Somerset, UK) in a touchdown PCR profile, in which the following condition was set: Initial denaturation at 94°C for 5 min followed by 10 cycles of touchdown 55–45°C, 20 s at 94°C, annealing for 20 s at 55°C (the annealing temperature for each cycle being reduced by 1°C per cycle) and extension for 30 s at 72°C. This was accompanied by 40 cycle of denaturation at 94°C for 30 s, annealing at 50°C for 30 s, elongation at 72°C for 45 s, and 10 min of final extension at 72°C. Amplified products were resolved in 3% agarose gel using 0.5 × TBE running buffer and images were analyzed in Quantity one software (Bio-Rad, CA 94547, USA).

### Genetic diversity and population structure analysis

The genetic diversity parameters such as major allele frequency, polymorphic information content (PIC), heterozygosity and alleles per locus were computed using PowerMarker v. 3.25 (Liu and Muse, 2005). Nei's gene diversity (h) and Shannon information index (I) were estimated in the sample using POPGENE v. 1.32. The model-based Bayesian approach was used to infer population structure with STRUCTURE v. 2.3.4 (Pritchard et al., 2000). The project was run with the admixture model and correlated allele frequency using burn in period of 20,000 and 200,000 Markov chain Monte Carlo (MCMC) replications. Five independent runs were performed with each *K* value ranging from 1 to 10. Evanno's Δ*k* value was calculated by using STRUCTURE HARVESTER program by processing the STRUCTURE results (Evanno et al., 2005). Concerning distance based clustering, DARwin v. 6.0.13 (Perrier and Jacquemoud-Collet, 2006) was employed to generate genetic distance (GD) matrix, which was then used to create dendrogram using unweighted neighbor joining. Factorial analysis was also performed with GD matrix created using DARwin software. We performed analysis of molecular variance (AMOVA) among and within subpopulations (assigned by STRUCTURE), implemented in GenAlEx (Peakall and Smouse, 2012). GenAlEx was also employed to cluster the genetic variation by performing principal coordinate analysis (PCoA).

### Trait mapping

The MTA for FW resistance was detected using TASSEL v. 2.1 (Bradbury et al., 2007) as described by Iqbal and Rahman (2017). The general linear model (GLM) and mixed linear model (MLM) based on Q matrix and Q+K matrix, respectively were employed to discover SSR markers associated with FW resistance. The structure analysis generated the Q matrix, while the relative kinship matrix was calculated by TASSEL software. Significant MTAs were declared at *P* ≤ 0.05 with corresponding *R*^2^ indicating the phenotypic variation explained by the MTA.

In parallel, a “coarse” linkage map was developed for the backcross population using IciMapping v. 4.1 with a minimum logarithm of odds (LOD) value of 2.5 (Meng et al., 2015). Segregation data was assembled for 75 SSR markers. By using 1% acetocarmine solution, segregation data on pollen fertility and sterility were reported earlier in this backcross population (Bohra et al., 2017). Kosambi mapping function was used to calculate genetic distances. Linkage map was drawn with MapChart v. 2.5 (Voorrips, 2002).

## Results

### Validation of hypervariable SSRs from pigeonpea genome

A set of 23,410 primer pairs was designed out of 309,052 SSRs identified in pigeonpea genome (Varshney et al., 2012). In the current analysis, 421 SSRs were selected based on length of the SSR tract (Class I or hypervariable) for amplification in pigeonpea lines. These 421 SSR markers were assayed on eight pigeonpea genotypes viz. Type 7, ICP 8863, PA 163A, ICPA 2089, AK 261322R, AK 261354R, AK 250189R, AK 250173R that are parents of different mapping populations (F_2_ and backcross) segregating for important traits such as *Fusarium* wilt and fertility restoration. Of the total 421 markers, 401 provided scorable amplicons and 217 markers generated polymorphic fragments. Twenty SSR markers failed to show amplification in any of the eight genotypes (Supplementary Table 3). In perfect SSR category (as defined by Weber, 1990), tetra- (NNNN), and penta-nucleotide (NNNNN) repeats showed 60% polymorphism followed by tri (NNN) and hexa-nucleotide (NNNNNN) repeats (Table 2).

**Table 2:** **Polymorphism of different SSR types across eight pigeonpea genotypes**.

**S. no**.	**SSR motif**	**No. of markers**	**No. of amplified markers**	**No. of polymorphic markers**	**Percent polymorphism**
**Perfect SSRs**					
1	Tri	335	325	184	56.6
2	Tetra	10	10	6	60.0
3	Penta	10	10	6	60.0
4	Hexa	16	16	4	25.0
Imperfect/complex SSRs	–	50	40	17	42.5
Total		421	401	217	

The polymorphism information content (PIC) of these 217 SSRs was in the range of 0.11–0.71 with an average 0.38 (Table 3). A total of 570 alleles were detected and the number of alleles per marker ranged from 2 to 6 with an average of 3. Similarly, the range of gene diversity lied between 0.12 and 0.75 with a mean of 0.44. The highest PIC value was shown by the marker CcGM20721, while the marker CcGM21506 generated maximum number of alleles across eight genotypes. We also attempted to find out a relation between the length of the SSR tract and the PIC value with the dataset comprising only perfect SSRs. However, a weak positive relationship (*r* = 0.1427 and *r*^2^ = 0.0204) could be inferred from the dataset.

**Table 3:** **Genetic diversity parameters based on analysis of eight pigeonpea genotypes with 217 SSR markers**.

**S. no**.	**Marker name**	**Major allele frequency**	**Allele number**	**Gene diversity**	**Heterozygosity**	**PIC**
1	CcGM00360	0.88	2	0.22	0.00	0.19
2	CcGM00931	0.50	2	0.50	0.00	0.38
3	CcGM01904	0.50	3	0.59	0.00	0.51
4	CcGM01991	0.75	2	0.38	0.00	0.30
5	CcGM02114	0.81	2	0.30	0.13	0.26
6	CcGM02585	0.38	3	0.66	0.00	0.58
7	CcGM03169	0.38	4	0.72	0.00	0.67
8	CcGM03373	0.50	4	0.65	0.38	0.59
9	CcGM03681	0.88	2	0.22	0.00	0.19
10	CcGM03809	0.88	2	0.22	0.00	0.19
11	CcGM04728	0.88	2	0.22	0.00	0.19
12	CcGM04943	0.88	2	0.22	0.00	0.19
13	CcGM05108	0.38	4	0.72	0.00	0.67
14	CcGM06350	0.56	4	0.60	0.13	0.55
15	CcGM06356	0.44	4	0.70	0.13	0.64
16	CcGM06568	0.50	3	0.59	0.00	0.51
17	CcGM06586	0.50	3	0.59	0.00	0.51
18	CcGM06587	0.56	2	0.49	0.13	0.37
19	CcGM07675	0.38	4	0.72	0.25	0.67
20	CcGM07873	0.44	3	0.65	0.13	0.57
21	CcGM08129	0.50	2	0.50	0.00	0.38
22	CcGM08668	0.88	2	0.22	0.00	0.19
23	CcGM08701	0.63	2	0.47	0.00	0.36
24	CcGM08896	0.56	4	0.62	0.13	0.57
25	CcGM09211	0.63	3	0.53	0.00	0.47
26	CcGM09457	0.38	4	0.69	0.00	0.63
27	CcGM09571	0.63	2	0.47	0.00	0.36
28	CcGM09707	0.63	2	0.47	0.00	0.36
29	CcGM10737	0.88	2	0.22	0.00	0.19
30	CcGM10832	0.88	2	0.22	0.00	0.19
31	CcGM10922	0.38	4	0.72	0.00	0.67
32	CcGM11620	0.75	2	0.38	0.00	0.30
33	CcGM11658	0.88	2	0.22	0.00	0.19
34	CcGM12037	0.50	3	0.63	0.00	0.55
35	CcGM12217	0.63	3	0.51	0.13	0.43
36	CcGM12275	0.44	4	0.68	0.13	0.62
37	CcGM12371	0.50	4	0.66	0.00	0.60
38	CCGM12576	0.63	2	0.47	0.00	0.36
39	CcGM12662	0.63	2	0.47	0.00	0.36
40	CcGM12694	0.75	2	0.38	0.00	0.30
41	CcGM13213	0.50	4	0.66	0.00	0.60
42	CcGM13254	0.88	2	0.22	0.00	0.19
43	CcGM13288	0.88	2	0.22	0.00	0.19
44	CcGM13428	0.88	2	0.22	0.00	0.19
45	CcGM13503	0.88	2	0.22	0.00	0.19
46	CcGM13505	0.88	2	0.22	0.00	0.19
47	CcGM13537	0.50	3	0.59	0.00	0.51
48	CcGM13637	0.88	2	0.22	0.00	0.19
49	CcGM13712	0.88	2	0.22	0.00	0.19
50	CcGM13766	0.88	2	0.22	0.00	0.19
51	CcGM13944	0.94	2	0.12	0.13	0.11
52	CcGM13964	0.38	3	0.66	0.00	0.58
53	CcGM14000	0.94	2	0.12	0.13	0.11
54	CcGM14057	0.69	3	0.48	0.13	0.43
55	CcGM14064	0.56	2	0.49	0.13	0.37
56	CcGM14109	0.88	2	0.22	0.00	0.19
57	CcGM14169	0.63	2	0.47	0.00	0.36
58	CcGM14207	0.50	3	0.63	0.25	0.55
59	CcGM14251	0.88	2	0.22	0.00	0.19
60	CcGM14252	0.38	4	0.72	0.00	0.67
61	CcGM14447	0.75	4	0.41	0.25	0.39
62	CcGM14463	0.69	2	0.43	0.38	0.34
63	CcGM14475	0.56	3	0.57	0.13	0.50
64	CcGM14521	0.63	3	0.53	0.00	0.47
65	CcGM14561	0.88	2	0.22	0.00	0.19
66	CcGM14613	0.63	2	0.47	0.00	0.36
67	CcGM14720	0.75	2	0.38	0.00	0.30
68	CcGM14753	0.50	3	0.63	0.00	0.55
69	CcGM14772	0.63	2	0.47	0.00	0.36
70	CcGM14937	0.63	2	0.47	0.00	0.36
71	CcGM14953	0.38	4	0.72	0.00	0.67
72	CcGM14962	0.88	2	0.22	0.00	0.19
73	CcGM15117	0.88	2	0.22	0.00	0.19
74	CcGM15129	0.63	3	0.53	0.00	0.47
75	CcGM15165	0.88	2	0.22	0.00	0.19
76	CcGM15232	0.75	2	0.38	0.00	0.30
77	CcGM15325	0.88	2	0.22	0.00	0.19
78	CcGM15449	0.69	4	0.49	0.13	0.46
79	CcGM15473	0.63	2	0.47	0.00	0.36
80	CcGM15508	0.63	4	0.56	0.00	0.52
81	CcGM15605	0.69	3	0.48	0.13	0.43
82	CcGM15710	0.75	3	0.40	0.13	0.35
83	CcGM15803	0.63	2	0.47	0.00	0.36
84	CcGM16001	0.75	3	0.41	0.00	0.37
85	CcGM16048	0.50	4	0.66	0.25	0.60
86	CcGM16285	0.75	2	0.38	0.00	0.30
87	CcGM16303	0.63	2	0.47	0.00	0.36
88	CcGM16323	0.38	3	0.66	0.00	0.58
89	CcGM16417	0.63	3	0.53	0.00	0.47
90	CcGM16529	0.69	3	0.48	0.13	0.43
91	CcGM16545	0.63	4	0.56	0.00	0.52
92	CcGM16546	0.50	2	0.50	0.25	0.38
93	CcGM16584	0.63	2	0.47	0.00	0.36
94	CcGM16612	0.81	2	0.30	0.13	0.26
95	CcGM16633	0.63	4	0.56	0.00	0.52
96	CcGM16723	0.88	2	0.22	0.00	0.19
97	CcGM16750	0.88	2	0.22	0.00	0.19
98	CcGM16772	0.50	4	0.66	0.13	0.62
99	CcGM16775	0.63	3	0.53	0.00	0.47
100	CcGM16799	0.63	2	0.47	0.00	0.36
101	CcGM16802	0.88	2	0.22	0.00	0.19
102	CcGM16858	0.75	3	0.41	0.00	0.37
103	CcGM16887	0.88	2	0.22	0.00	0.19
104	CcGM17051	0.75	3	0.41	0.00	0.37
105	CcGM17100	0.63	3	0.53	0.00	0.47
106	CcGM17150	0.38	3	0.66	0.13	0.59
107	CcGM17154	0.50	3	0.59	0.00	0.51
108	CcGM17176	0.75	3	0.41	0.00	0.37
109	CcGM17379	0.63	3	0.53	0.25	0.47
110	CcGM17425	0.75	3	0.41	0.00	0.37
111	CcGM17438	0.75	2	0.38	0.00	0.30
112	CcGM17475	0.63	3	0.53	0.00	0.47
113	CcGM17543	0.81	2	0.30	0.13	0.26
114	CcGM17611	0.50	4	0.66	0.00	0.60
115	CcGM17614	0.38	4	0.69	0.00	0.63
116	CcGM17620	0.69	2	0.43	0.13	0.34
117	CcGM17648	0.50	2	0.50	0.00	0.38
118	CcGM17657	0.75	2	0.38	0.00	0.30
119	CcGM17797	0.50	3	0.59	0.00	0.51
120	CcGM17816	0.50	3	0.59	0.00	0.51
121	CcGM17845	0.63	2	0.47	0.00	0.36
122	CcGM17946	0.50	3	0.59	0.00	0.51
123	CcGM17970	0.88	2	0.22	0.00	0.19
124	CcGM18008	0.38	3	0.66	0.00	0.58
125	CcGM18041	0.63	2	0.47	0.00	0.36
126	CcGM18042	0.63	3	0.53	0.00	0.47
127	CcGM18196	0.88	3	0.23	0.25	0.21
128	CcGM18273	0.88	2	0.22	0.00	0.19
129	CcGM18291	0.63	2	0.47	0.00	0.36
130	CcGM18384	0.63	2	0.47	0.00	0.36
131	CcGM18517	0.75	2	0.38	0.00	0.30
132	CcGM18538	0.63	2	0.47	0.00	0.36
133	CcGM18599	0.88	2	0.22	0.00	0.19
134	CcGM18676	0.75	3	0.41	0.00	0.37
135	CcGM18681	0.75	3	0.41	0.00	0.37
136	CcGM18684	0.50	4	0.66	0.00	0.60
137	CcGM18785	0.75	3	0.41	0.00	0.37
138	CcGM18867	0.69	3	0.48	0.13	0.43
139	CcGM18876	0.56	3	0.54	0.13	0.45
140	CcGM18923	0.56	3	0.57	0.25	0.50
141	CcGM19108	0.50	2	0.50	0.00	0.38
142	CcGM19123	0.38	3	0.66	0.25	0.58
143	CcGM19136	0.50	2	0.50	0.00	0.38
144	CcGM19144	0.75	3	0.41	0.00	0.37
145	CcGM19152	0.81	3	0.32	0.13	0.29
146	CcGM19217	0.38	4	0.68	0.13	0.62
147	CcGM19277	0.63	2	0.47	0.00	0.36
148	CcGM19285	0.63	2	0.47	0.50	0.36
149	CcGM19325	0.75	2	0.38	0.00	0.30
150	CcGM19413	0.38	4	0.69	0.25	0.63
151	CcGM19472	0.69	3	0.48	0.13	0.43
152	CcGM19565	0.63	4	0.56	0.25	0.52
153	CcGM19566	0.88	2	0.22	0.00	0.19
154	CcGM19652	0.75	2	0.38	0.00	0.30
155	CcGM19653	0.63	2	0.47	0.00	0.36
156	CcGM19705	0.38	3	0.66	0.00	0.58
157	CcGM19861	0.50	2	0.50	0.00	0.38
158	CcGM19876	0.81	3	0.32	0.13	0.29
159	CcGM19907	0.88	2	0.22	0.00	0.19
160	CcGM19934	0.63	3	0.53	0.00	0.47
161	CcGM20007	0.94	2	0.12	0.13	0.11
162	CcGM20110	0.88	2	0.22	0.00	0.19
163	CcGM20155	0.88	2	0.22	0.00	0.19
164	CcGM20163	0.88	2	0.22	0.00	0.19
165	CcGM20190	0.75	2	0.38	0.25	0.30
166	CcGM20208	0.88	2	0.22	0.00	0.19
167	CcGM20296	0.75	3	0.41	0.00	0.37
168	CcGM20342	0.56	3	0.59	0.13	0.52
169	CcGM20404	0.63	2	0.47	0.00	0.36
170	CcGM20407	0.63	2	0.47	0.00	0.36
171	CcGM20512	0.38	4	0.69	0.00	0.63
172	CcGM20603	0.88	2	0.22	0.00	0.19
173	CcGM20620	0.44	4	0.65	0.13	0.58
174	CcGM20721	0.38	5	0.75	0.00	0.71
175	CcGM20775	0.88	2	0.22	0.00	0.19
176	CcGM21015	0.38	3	0.66	0.00	0.58
177	CcGM21038	0.75	3	0.41	0.00	0.37
178	CcGM21044	0.63	3	0.53	0.00	0.47
179	CcGM21056	0.75	2	0.38	0.00	0.30
180	CcGM21072	0.63	2	0.47	0.00	0.36
181	CcGM21079	0.75	3	0.41	0.00	0.37
182	CcGM21170	0.88	2	0.22	0.00	0.19
183	CcGM21174	0.88	2	0.22	0.25	0.19
184	CcGM21321	0.50	3	0.63	0.25	0.55
185	CcGM21476	0.50	2	0.50	0.00	0.38
186	CcGM21502	0.88	2	0.22	0.00	0.19
187	CcGM21506	0.50	6	0.70	0.13	0.67
188	CcGM21628	0.75	2	0.38	0.00	0.30
189	CcGM21644	0.88	2	0.22	0.00	0.19
190	CcGM21693	0.44	5	0.69	0.88	0.64
191	CcGM21774	0.88	2	0.22	0.00	0.19
192	CcGM21816	0.63	2	0.47	0.00	0.36
193	CcGM21910	0.38	3	0.66	0.00	0.58
194	CcGM22072	0.88	2	0.22	0.00	0.19
195	CcGM22116	0.75	3	0.41	0.00	0.37
196	CcGM22151	0.50	3	0.59	0.00	0.51
197	CcGM22222	0.50	2	0.50	0.00	0.38
198	CcGM22227	0.88	2	0.22	0.00	0.19
199	CcGM22341	0.50	3	0.63	0.00	0.55
200	CcGM22418	0.38	4	0.72	0.00	0.67
201	CcGM22436	0.69	3	0.48	0.13	0.43
202	CcGM22440	0.50	3	0.59	0.00	0.51
203	CcGM22559	0.81	2	0.30	0.13	0.26
204	CcGM22570	0.75	2	0.38	0.00	0.30
205	CcGM22747	0.50	3	0.59	0.25	0.51
206	CcGM22805	0.88	2	0.22	0.00	0.19
207	CcGM22850	0.94	2	0.12	0.13	0.11
208	CcGM22990	0.63	2	0.47	0.00	0.36
209	CcGM22992	0.88	2	0.22	0.00	0.19
210	CcGM23005	0.63	2	0.47	0.00	0.36
211	CcGM23062	0.88	2	0.22	0.00	0.19
212	CcGM23131	0.50	3	0.59	0.00	0.51
213	CcGM23176	0.75	2	0.38	0.00	0.30
214	CcGM23262	0.75	2	0.38	0.00	0.30
215	CcGM23321	0.88	2	0.22	0.00	0.19
216	CcGM23354	0.75	2	0.38	0.00	0.30
217	CcGM23371	0.63	2	0.47	0.00	0.36

In the case of pairwise polymorphism among parental lines of mapping populations a total 179 SSR markers showed polymorphism at least within one crossing combination (Supplementary Table 3). The number of polymorphic markers for parental combinations ranged from 39 (PA 163A × AK 261322R) to 89 (Type 7 × ICP 8863).

### The genetic diversity and population structure of cultivated pigeonpea

**Figure 1:**
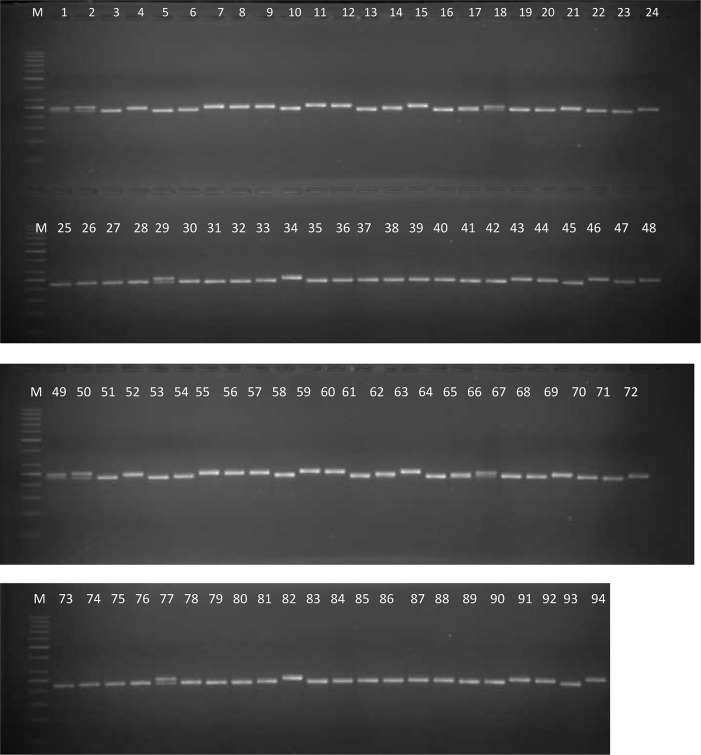
**A representative gel image showing SSR profiles of 94 pigeonpea genotypes using SSR marker CcGM22990**. Lane M, 100 bp standard DNA ladder; lanes 1–94, genotypes.

Keeping in view the high quality marker profiling patterns and PIC values, a subset of 60 markers was selected from the 217 polymorphic markers for analyzing 94 genotypes. As a result, a total of 233 alleles were obtained across the genotypes. The average values for PIC and number of alleles per marker were 0.50 and 3.88, respectively. A representative image illustrating the SSR fingerprints of the 94 genotypes using the marker CcGM22990 has been shown in Figure 1. Though only four landraces were used in the current analysis, we analyzed 15 plants of these landraces and two varieties (IPA 203 and Narendra Arhar 1) using CcGM18684 to evaluate the within-genotype variability. No within genotype variability and heterozygosity was observed in the varieties. By contrast, the three landraces showed two alleles each and variable level of heterozygosity [0.06 (Banda Palera and Allahabad Local) and 0.2 (JBP-13)] (Supplementary Figure 1).

**Figure 2:**
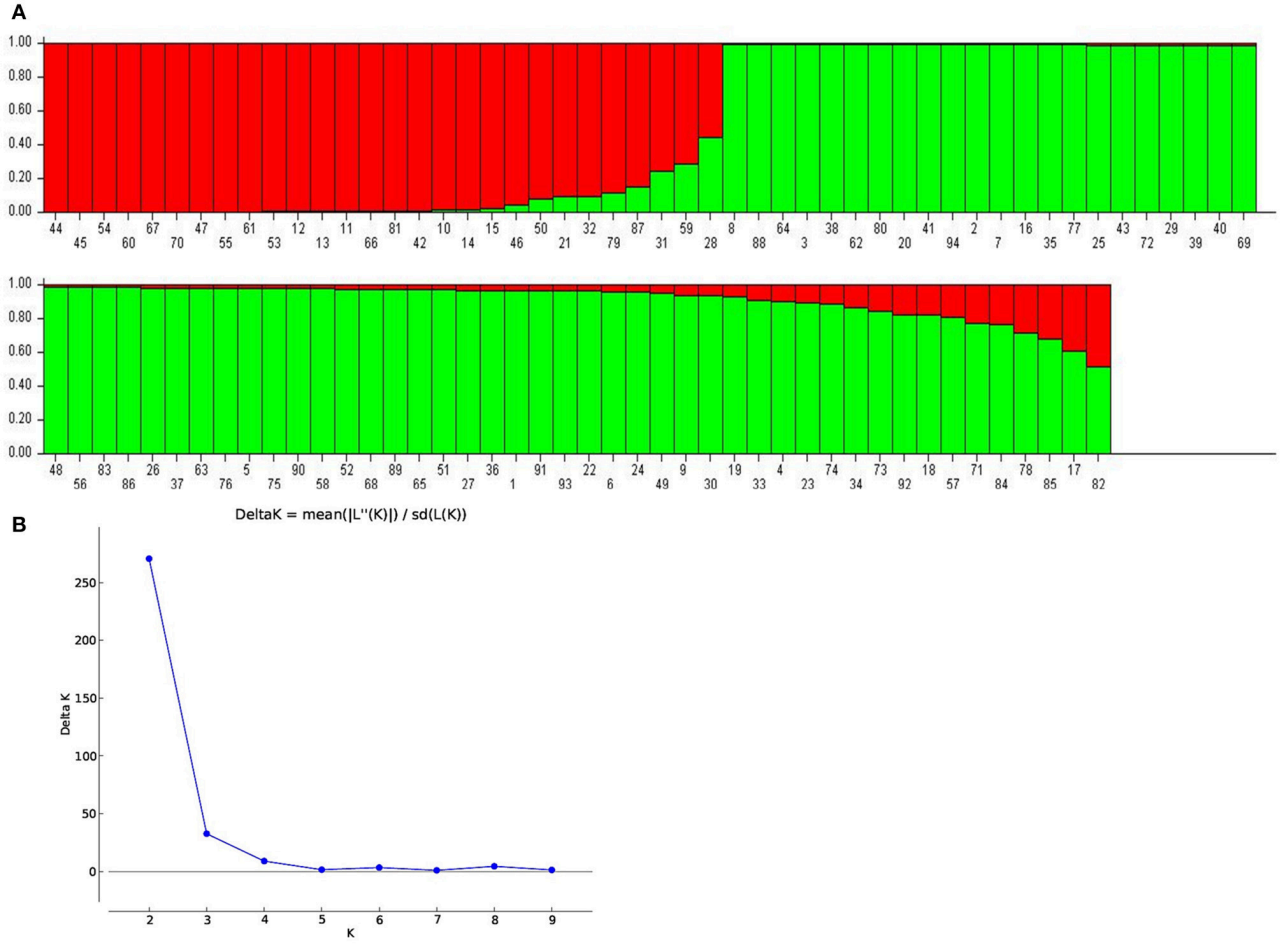
**(A)** Population structure inferred from 60 SSR markers. Each genotype is represented by a vertical bar carrying K colored segments which indicates the estimated membership proportion to the K cluster (*K* = 2). Serial numbers are assigned according to Supplementary Table 1. **(B)** The true value of K was obtained following the delta K method of Evanno et al. (2005).

To examine the genetic variations identified through 60 SSR markers across 94 genotypes, model- as well as distance-based approaches were used. In model based clustering, five independent runs were performed using STRUCTURE programme with *K* values ranging from 1 to 10. The maximum delta K (*ad-hoc* quantity) was reached at *K* = 2 suggesting presence of two subpopulations in the collection (Figure 2). Of the total 94 genotypes, 28 belonged to subpopulation 1, while remaining 66 corresponded to subpopulation 2 in STRUCTURE analysis. With a few exceptions, the subpopulation 2 contained short- and medium-duration pigeonpea, whereas the genotypes with longer maturity duration were in subpopulation 1. The mean Nei's gene diversity was found to be 0.50 and 0.56 for subpopulation 1 and 2, respectively. Similarly, mean values for Shannon information index for subpopulation 1 and 2 were 0.91 and 1.04, respectively. Overall, the average values of Nei's gene diversity and Shannon information index were 0.58 and 1.089, respectively.

**Figure 3:**
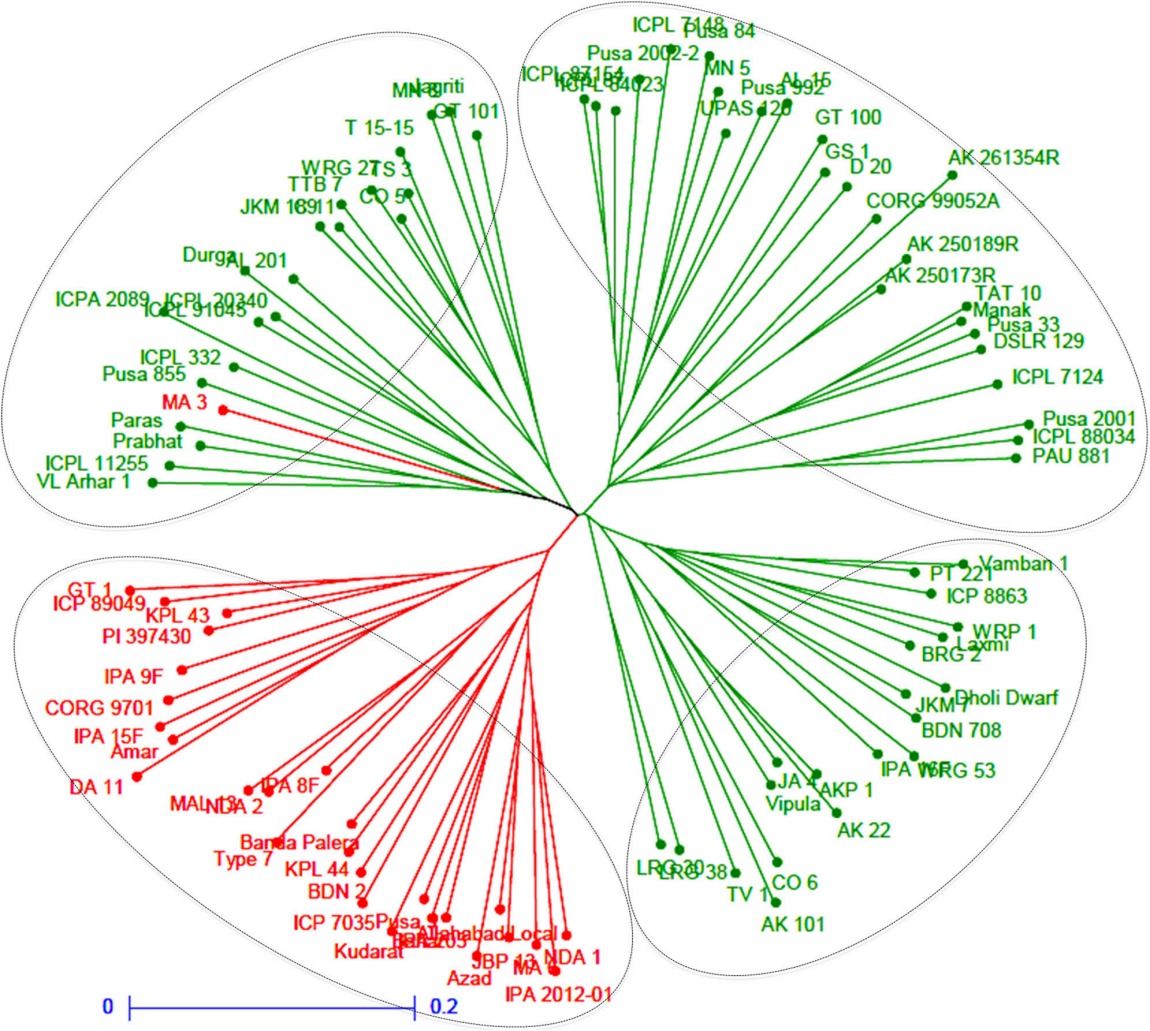
**Neighbor joining tree based on GD matrix of 94 pigeonpea genotypes**. The two colors correspond to the two subgroups assigned by the STRUCTURE.

**Figure 4:**
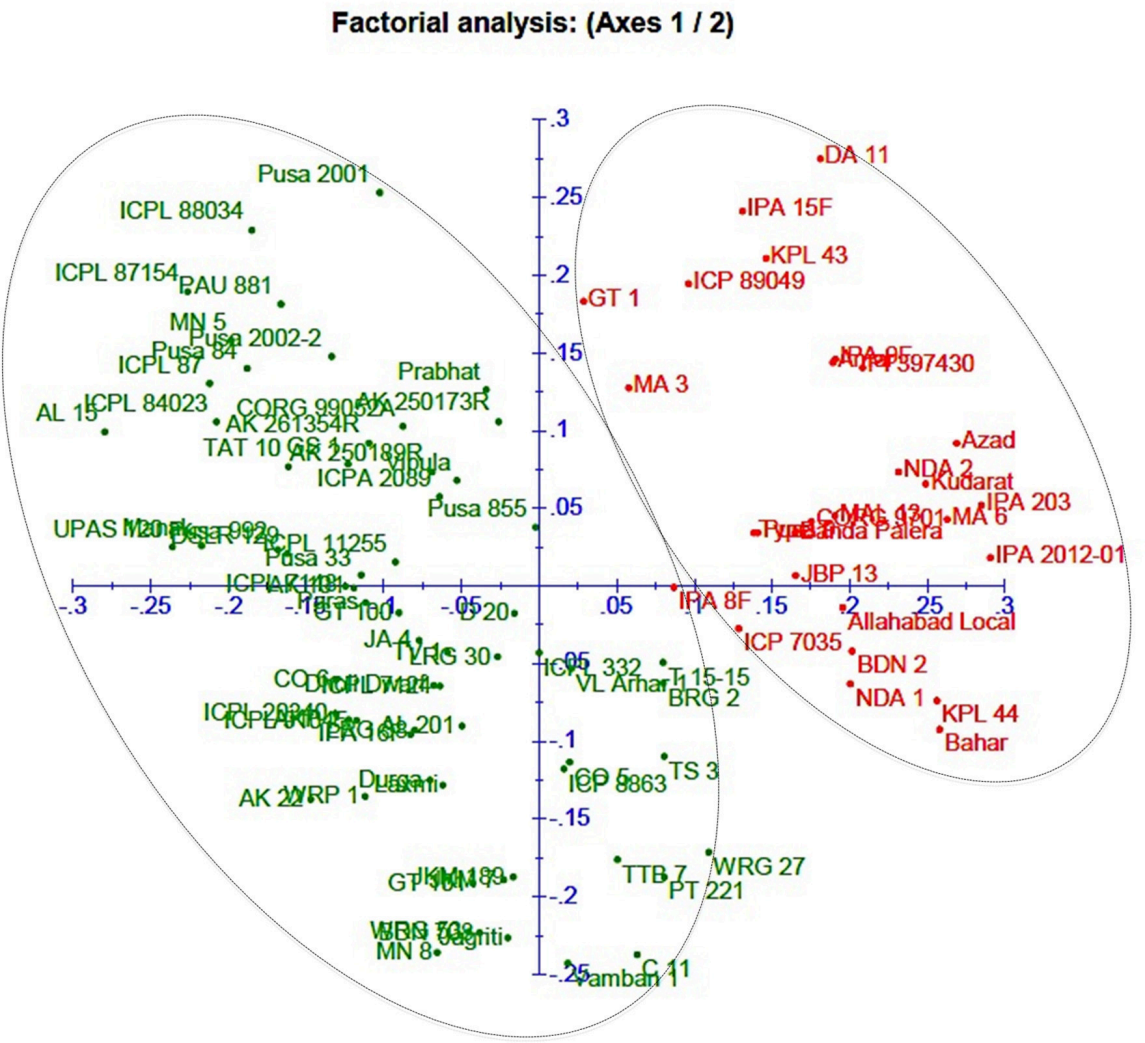
**Factorial analysis of the 94 pigeonpea genotypes based on 60 SSR markers**.

**Figure 5:**
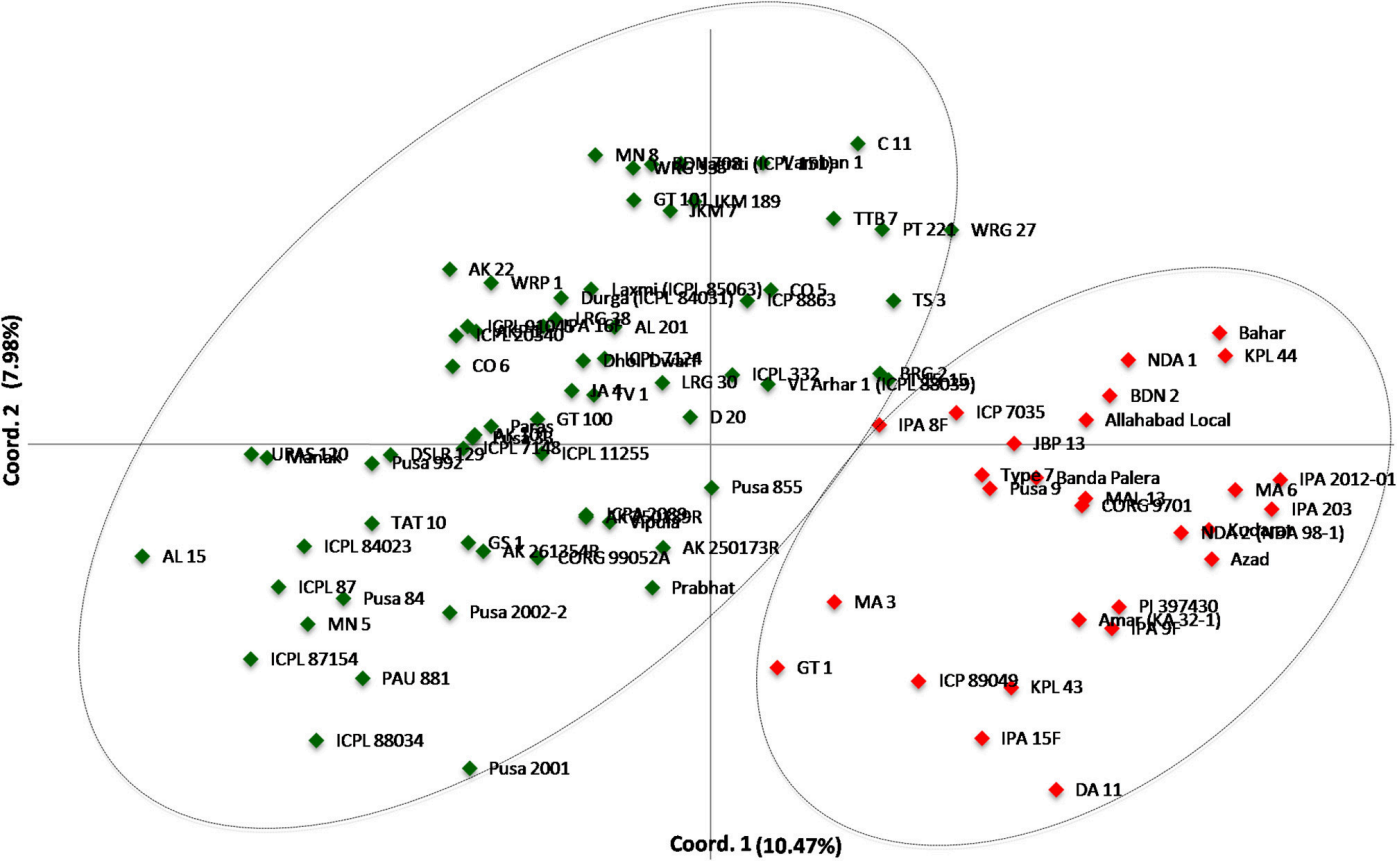
**Scatter plot of the PC1 and PC2 of the 94 pigeonpea genotypes**. The PC1 explains 10.47% of the variance, while the PC2 accounts for 7.98%.

A neighbor-joining tree was constructed based on genetic distance matrix, which suggested existence of four clusters (Figure 3). The factorial analysis was also undertaken to offer an overall representation of the diversity in the studied panel. Interestingly, a close agreement was observed between the results arising from STRUCTURE and factorial analysis. The subpopulation 2 of STRUCTURE was contained primarily in quadrants II and III of factorial analysis (Figure 4). On the other hand, quadrant I contained genotypes that were assigned to subpopulation 1 in STRUCTURE analysis. Similarly, two clusters were also obtained in PCoA with the PC1 and PC2 explaining 10.47 and 7.98% of the variance, respectively (Figure 5). Further, the total genetic variation was partitioned by using AMOVA based on PhiPT-values, which accounted majority of the genetic variation to within groups (89%) in comparison to the variation observed between the groups (11%) (Table 4).

**Table 4:** **Summary of AMOVA**.

**Source**	**df**	**SS**	**MS**	**Est. Var**.	**%**
Among Pops	1	374.66	374.659	7.951	11%
Within Pops	92	5707.93	62.043	62.043	89%
Total	93	6082.60		69.994	100%

### Trends in decadal and zonal genetic diversity in pigeonpea cultivars

The marker genotyping data on 59 varieties released in India (a subset of above mentioned 94 genotypes) during 1975 and 2015 were also examined to fathom trend of genetic diversity according to both decadal period and zone. Four subgroups could be created each for decadal and zonal study. A pairwise comparison among different groups suggested the maximum GD between the varietal groups pertaining to decade 1 (1975–1985) and decade 4 (2005–2015), while the least GD was shown between the groups decade 1 (1975–1985) and decade 2 (1986–1995). On the other hand, the least GD was found between CZ and SZ groups in contrast to the highest GD between NWPZ and NEPZ (Table 5). The data pertaining to means of gene diversity and number of alleles among these groups are shown in Table 6. Concerning decadal trend, the estimates of gene diversity and average number of alleles per locus did not show large difference. In a similar manner, average values for gene diversity and allele count remain almost similar among the four groups obtained based on their suitability to different zones.

**Table 5:** **Nei's unbiased measures of genetic distance**.

**(A) Among decadal groups of Indian pigeonpea cultivars**.				
	**1975–1985**	**1986–1995**	**1996–2005**	**2005–2015**
1975–1985	–			
1986–1995	0.0313	–		
1996–2005	0.0716	0.0385	–	
2005–2015	0.073	0.0443	0.0627	–
**(B) Among zonal groups of Indian pigeonpea cultivars**.				
	**NWPZ**	**SZ**	**CZ**	**NEPZ**
NWPZ	–			
SZ	0.1281	–		
CZ	0.1317	0.0247	–	
NEPZ	0.2472	0.1866	0.1542	–

**Table 6:** **Trend in genetic diversity of Indian pigeonpea cultivars as reflected from decadal periods and zones**.

**Decadal genetic diversity**				**Zonal genetic diversity**			
**Decade**	**Sample size**	**Average number of alleles per locus**	**Gene diversity**	**Zone**	**Sample size**	**Average number of alleles per locus**	**Gene diversity**
1975–1985	15	3.51	0.54	NWPZ	13	3.15	0.53
1986–1995	17	3.56	0.56	SZ	19	3.38	0.52
1996–2005	18	3.33	0.53	CZ	15	3.38	0.52
2005–2015	9	2.95	0.51	NEPZ	12	3.11	0.49

### Hybridity testing of CMS based hybrids

**Figure 6:**
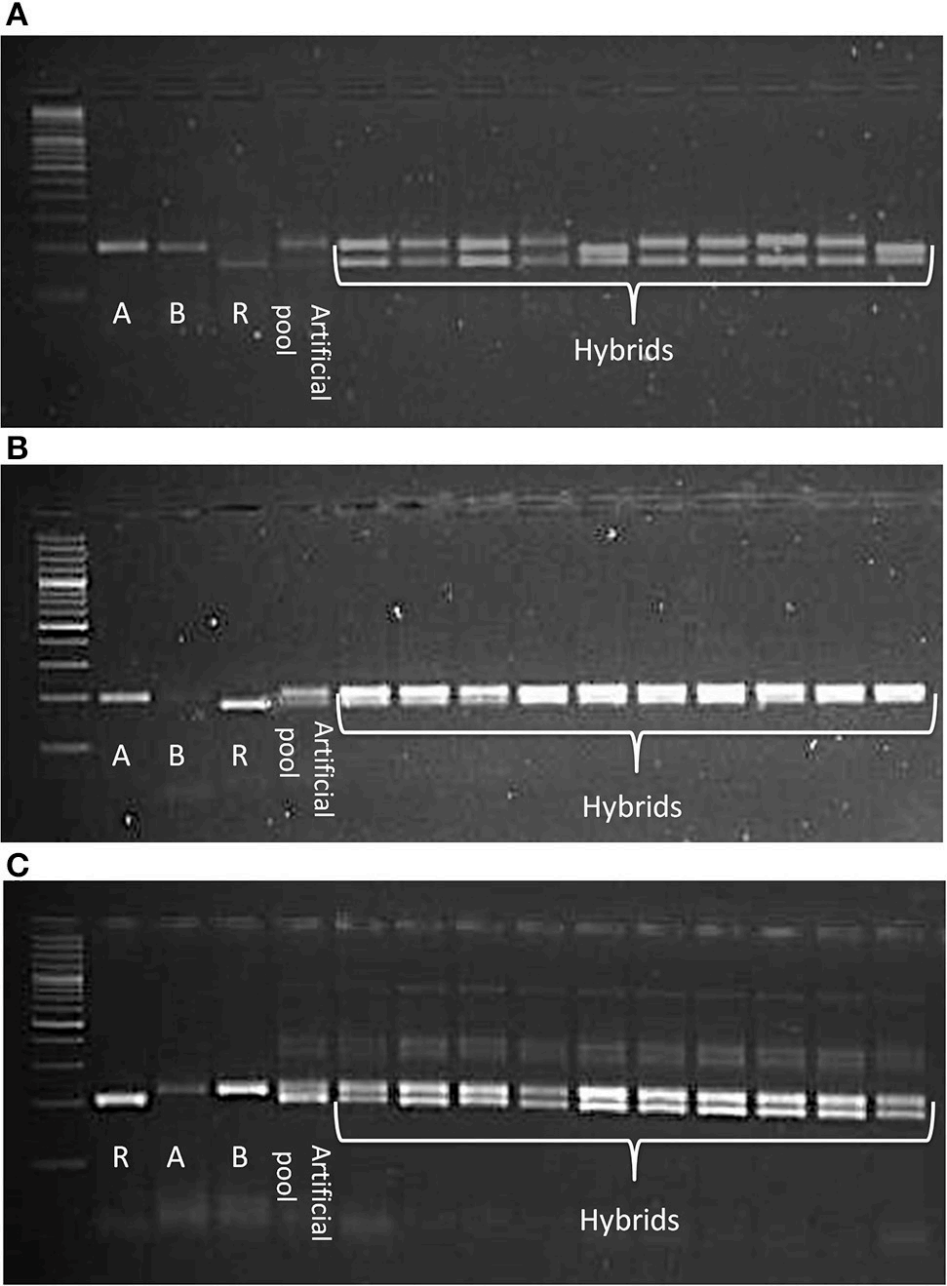
**Gel images illustrating utility of CcGM markers in genetic purity testing of the CMS hybrids**. Ten plants each of three CMS hybrids were selected for SSR analysis. Artificial pool was constructed by mixing genomic DNA of two parents of the respective hybrid. Shown are the SSR profiles of **(A)** IPAH 16-06 using CcGM16529, **(B)** IPAH 16-07 using CcGM16772, and **(C)** IPH 15-03 using CcGM17648.

CMS technology has emerged as a promising means to deliver noticeable yield gains in pigeonpea. The CMS-based hybrid breeding involves three parental genotypes: male sterile (A) line, its isogenic line (B) line and fertility restorer (R) line. Ten SSR markers were tested in three CMS hybrids (IPAH 16-06, IPAH 16-07, and IPH 15-03) and respective parents i.e., A, B, and R lines. These hybrids are being tested in evaluation trails at different locations in India under all India coordinated research projects on pigeonpea (AICRPP) and consortium research platform on hybrid technology (CRPHT) schemes. The SSR markers that generated monomorphic fragments between A and B lines and polymorphic fragments between the A and R lines were selected for testing the genetic purity of the respective hybrids. Ten random samples were taken from each hybrid for SSR analysis. As a result, sets of SSRs that were found promising for the molecular characterization of the three CMS hybrids included three markers (CcGM16529, CcGM16633, CcGM16772) for IPAH 16-06; three markers (CcGM16772, CcGM17150, CcGM23176) for IPAH 16-07 and four markers (CcGM18291, CcGM17648, CcGM12217, CcGM16417) for IPH 15-03 (Figure 6).

### Trait mapping

#### Association analysis

The wilt incidence data and genotyping data of 60 SSRs on 40 pigeonpea genotypes were analyzed to detect SSR markers associated with the FW resistance. Interestingly, only one SSR marker (CcGM03681) could be declared as linked with the FW resistance using both GLM and MLM. In both models, the phenotypic variance (*R*^2^) accounted to the SSR CcGM03681 was found to be 16.4%.

#### Genetic analysis of a backcross population and mapping of Rf locus

Marker screening of a total of 824 SSRs (421 CcGMs, 261 HASSRs, 60 CcMs, 12 CZs, 10 CCBs, and 60 ASSRs) between the mapping parents (ICPL 88039A and AK 250189R) provided 115 polymorphic markers. Segregation data were assembled for 75 SSR markers and subjected to goodness-of-fit test. As a result, 55 SSRs showed Mendelian segregation of 1:1, while remaining 20 markers deviated from the ratio (Supplementary Table 4). The phenotypic segregation ratio of 1 (fertile): 1 (sterile) as evident from pollen fertility assay in this population showed possibility of one dominant gene for fertility restoration (Bohra et al., 2017). We could place 35 markers on to the coarse linkage map, and the *Rf* locus could be assigned at 39 cM with the marker CcGM08896 located on Scaffold130507 in the pigeonpea genome (Supplementary Figure 2).

## Discussion

Whole genome sequencing of Asha (ICPL 87119) has offered access to genome wide genetic variants such as SSRs and SNPs for their widespread applications in genomics and breeding. More recently, clustering patterns emerged by applying genome-wide SSR markers in rice showed closer agreement with the pedigree when compared with the SNP markers, thus highlighting the benefits associated with SSR markers (Gonzaga et al., 2015). The relevance of hypervariable SSR markers to plant breeding is well described in various crops including rice (Singh et al., 2010; Narshimulu et al., 2011).

We selected a set of 421 hyper-variable SSR markers for the present work. The validation success rate of 95.24% of the current investigation was comparable to earlier reported by Bohra et al. (2011) using BAC end sequence (BES)-derived SSR markers (96.48%), however the percentage was greater than reported in previous SSR-based studies in pigeonpea (Raju et al., 2010; Saxena et al., 2010a,b; Dutta et al., 2011; Singh et al., 2012). In a similar way, the percent polymorphism (54.11%) reported here was also greater than found earlier in pigeonpea. For instance, the percent polymorphism shown by SSR markers was reported to be 15% (Raju et al., 2010), 28.4% (Bohra et al., 2011), 47.94% (Odeny et al., 2009), and 48.71% (Odeny et al., 2007). Considering only hypervariable SSRs, the level of DNA polymorphism detected here was higher than found previously in pigeonpea (40.8%: Singh et al., 2012, 41.1%: Dutta et al., 2013). Considering an earlier study (Singh et al., 2012) where complex type SSR markers showed least polymorphism, present study focused majorly on the perfect SSRs (88%). This underscores the significance of the SSR markers experimentally validated in the present investigation.

As can be inferred from Table 7, the average PIC value of the current analysis corroborates with previously reported mean PIC values for SSR markers in pigeonpea, however the allele count was somewhat lower. The reason that explains smaller mean value for alleles per marker is possibly that the current study considers only *C. cajan* genotypes for diversity estimation. Incorporation of genotypes in the panel belonging to different species might lead to an increased number of alleles given by a particular DNA marker (Odeny et al., 2009; Singh D. et al., 2016). However, the present panel offers realistic PIC values in concern with pigeonpea breeding in India.

**Table 7:** **An overview of SSR-based diversity analyses in pigeonpea**.

**S. No**.	**Polymorphic markers**	**Panel size**		**PIC value count**	**Average allele**	**References**
		**Cultivated**	**Wild**			
1	10	12		–	3.1	Burns et al., 2001
2	19	15	9	0.39	3.4	Odeny et al., 2007
3	35	24		0.41	3.1	Odeny et al., 2009
4	13	32	8	0.32	5.5	Saxena et al., 2010a
5	23	32		0.43	2.7	Saxena et al., 2010b
5	41	159		0.41	3.1	Saxena et al., 2010c
6	15	40		0.40	4.0	Raju et al., 2010
8	842	21	1	0.57	5.65	Bohra et al., 2011
9	20	22	8	0.63	6.25	Dutta et al., 2011
10	24	40	8	0.47	2.71	Dutta et al., 2013
11	48	40		0.30	5.58	Njung'e et al., 2016

A lower genetic diversity in the cultivated pigeonpea as illustrated through Nei's gene diversity is not surprising, and is congruent with previous SSR-based diversity studies in pigeonpea. In addition to SSR marker, the narrow genetic base of the domesticated pigeonpea was also evident from analyses based on other DNA marker systems such as RAPD (Ratnaparkhe et al., 1995), RFLP (Nadimpalli et al., 1993), AFLP (Panguluri et al., 2006), DArT (Yang et al., 2006), ISR (Kudapa et al., 2012), and SNP (Kassa et al., 2012). The SSR analysis of the 15 plants from landraces and varieties suggested existence of within-genotype variability in landraces. This in turn highlights the need for pooling DNA from 10 to 15 plants of a single genotype in diversity analysis particularly in case of landraces that harbor greater level of genetic variation/heterogeneity (Brondani et al., 2006). Preference for uniform varieties in place of landraces has played important role in the loss of crop genetic diversity (van de Wouw et al., 2010).

Limited studies have been performed so far in pigeonpea that examine the population structure of the *C. cajan*. The existence of two major subpopulations in the sample was strongly supported by a correspondence between results arising from both model and distance based clustering methods. Further, the AMOVA based partitioning of the total variance also found resemblance with the patterns seen earlier in pigeonpea (Kassa et al., 2012) and also in other food legume crops such as lentil (Khazaei et al., 2016).

With a slight deviation, the subgroup I harbored long duration pigeonpea genotypes that are grown exclusively in north eastern plain zone (NEPZ) in India, particularly states like Bihar and Uttar Pradesh. A careful examination of pedigree of the genotypes contained in subgroup I revealed a popular variety Bahar as one of the parents (Singh I. et al., 2016). The pattern of grouping of genotypes, in particular the genotypes with long maturity duration showed similarity with the previous findings (Choudhury et al., 2008; Singh et al., 2013). We also attempted to dissect the genetic diversity among Indian cultivars based on their release year and area of adaptation. Unlike major staple crops like rice (Choudhary et al., 2013) and wheat (Mir et al., 2011), no significant trends in genetic diversity could emerge from the analysis of Indian pigeonpea varieties. However, a near constant (albeit moderate) genetic diversity among Indian varieties over the last 50 years demands excavation and recruitment of diverse alleles from the exotic or wild germplasm pool.

In addition to varietal improvement, harnessing hybrid vigor using CMS technology has emerged as a promising means to overcome the problem of yield stagnation in pigeonpea (Bohra et al., 2016). Supply of genetically pure seeds remains central to the successful hybrid breeding programme (Saxena et al., 2010c). Molecular markers, particularly SSRs, are immensely useful in order to ensure the genetic purity of the seeds of hybrids and its parents (Saxena et al., 2010c; Bohra et al., 2015). Here we identify sets of robust SSR markers that offer a rapid and cost-effective genomic tools for genetic purity testing of the representative lot. Such DNA markers would serve as a great supplement to the seed certification program and hybrid identification. In parallel, we also attempted to show the utility of these SSR markers in trait mapping using association as well as bi-parental linkage analyses. As a result of association mapping one SSR marker showed significant association with the FW resistance, while bi-parental linkage analysis established association of the *Rf* locus (restoring A_2_-CMS) with the marker CcGM08896. Earlier, Singh et al. (2013) analyzed 36 pigeonpea genotypes using SSR markers and found MTAs explaining the phenotypic variation in the range of 23–56%. To best of our knowledge, this represents the first study to report molecular mapping of *Rf* locus responsible for fertility restoration in A_2_-CMS. Earlier, QTLs for A_4_-CMS restoration were reported based on the analysis of three segregating F_2_ populations (Bohra et al., 2012). However, the MTAs detected in the present study are of very preliminary type and need extensive investigations including validation in diverse genetic backgrounds and multi-environmental evaluation to reach valid conclusion.

In summary, here we provide a set of 401 validated SSR markers and implicate them in the assessment of genetic variation and population structure of pigeonpea collection, genetic purity testing of CMS hybrids and bi-parental linkage and association analyses. A greater polymorphism percentage coupled with the consistent amplification patterns renders these SSR markers highly suitable for genotyping pigeonpea using simple lab equipment. These are important molecular tools that will certainly help pigeonpea research community for various molecular applications including marker-assisted selection. The clustering patterns resulting from model and distance based approaches will guide pigeonpea breeders for selection of the most diverse parental lines in future breeding programmes. Once validated in diverse genetic backgrounds, the MTAs detected here will pave the way for fine mapping/MAS of the loci controlling FW resistance and A_2_-CMS fertility restoration. Similarly, the genome wide SSR markers would facilitate background selection while practicing marker assisted back crossing. Also, the genetic populations reported here represent valuable genetic resources that will allow trait mapping and subsequent targeted trait improvement in pigeonpea.

## Author contributions

AB and RKV conceived the idea and planned experiments; RJ, GP, and AM performed genotyping; AB, GP, PGP, and DS analyzed the data; IPS, FS, and RKM contributed germplasm; AB, RKV, RKS, and NPS participated in writing and finalizing the manuscript. All authors have read the manuscript.

### Conflict of interest statement

The authors declare that the research was conducted in the absence of any commercial or financial relationships that could be construed as a potential conflict of interest.
