# Supplementary material for: New Hypervariable SSR Markers for Diversity Analysis, Hybrid Purity Testing and Trait Mapping in Pigeonpea [Cajanus cajan (L.) Millspaugh]
Source: Front Plant Sci. 2017 Mar 31;8:377. doi: 10.3389/fpls.2017.00377 (PMC5374739; doi:10.3389/fpls.2017.00377)
Supplement: Supplementary file 1 [file Table1.DOC]

**Supplementary Table 1: Salient features of 94 genotypes used in diversity analysis**

| **S. No.** | **Genotype** | **Pedigree** | **Area of adaptation** | **Salient features** |
| --- | --- | --- | --- | --- |
| 1 | Abhaya (ICPL 332) | Selection of ICP 1903-E-1-4EB | South Zone (SZ) | Indeterminate, semi-spreading, medium duration |
| 2 | AK 101 | - | Maharashtra | Indeterminate, erect, tall, dark brown seed colour |
| 3 | AK 22 | - | Maharashtra |  |
| 4 | AK 250173R | AK 101 × *Cajanus volubilis* | Maharashtra | Indeterminate, semi spreading, early maturity |
| 5 | AK 250189R | AK 101 × *C. volubilis* | Maharashtra | Indeterminate, semi spreading, early maturity |
| 6 | AK 261354R | AK 101 × *C. volubilis* | Maharashtra | Indeterminate, semi spreading, early maturity |
| 7 | AKP 1 | - | Maharashtra | Determinate, erect, dark brown and bold seed |
| 8 | AL 15 | Selection of P 8-9 | Punjab | Determinate, early-maturing, small seeded |
| 9 | AL 201 | AL 16 × EE 76 | Punjab | Indeterminate, compact and early maturing |
| 10 | Allahabad Local | Local selection from Allahabad, Uttar Pradesh | Uttar Pradesh (UP) | Indeterminate, semi-spreading, late maturing, resistant to sterility mosaic disease (SMD), small seeded |
| 11 | Amar (KA 32-1) | Selection of Bahar | UP | Compact, long duration, resistant to SMD |
| 12 | Azad | Bahar × KPBR 80-1 | UP | Indeterminate, long duration |
| 13 | Bahar | Selection from Motihari in Bihar | North Eastern plain zone (NEPZ) | Compact plant type, long duration, resistant to SMD |
| 14 | Banda Palera | Selection from Palera village of Banda district, Uttar Pradesh | UP | Indeterminate, spreading, red seeded, resistant to SMD and FW |
| 15 | BDN 2 | Sel. of BOR II | Maharashtra | Indeterminate, semi-spreading |
| 16 | BDN 708 | BDN × ICPL 87119 | Maharashtra | Moderately resistant to FW and SMD |
| 17 | BRG 2 | Selection From Nalaman gala local | Karnataka | Indeterminate, semi-spreading |
| 18 | C 11 | Selection of Sangareddy | Central Zone (CZ) | Indeterminate, profuse branching, brown seed, mid-long duration, tolerant to FW |
| 19 | CO 5 | Selection of CO 1 | Tamil Nadu | Indeterminate, semi-spreading, early, bushy, resistant to SMD |
| 20 | CO 6 | Mutant of SA 1 | Tamil Nadu | Semi-spreading, indeterminate |
| 21 | CORG 9701 | Selection from the genotype PB 9825 | SZ | Indeterminate, semi-spreading, early maturity |
| 22 | CORG 99052A | *Cajanus* *scarabaeoides* × CORG 9060 | Tamil Nadu | Indeterminate, compact, early-maturing, orange seeded |
| 23 | D 20 | - |  | Indeterminate, semi-spreading, early-maturing, white seed color |
| 24 | Dholi Dwarf | Local selection from Dholi, Bihar | Bihar | Determinate, semi-spreading, late maturing, resistant to SMD and tolerant to FW |
| 25 | DSLR 129 | - |  | Indeterminate, semi-spreading, early maturing |
| 26 | Durga (ICPL 84031) | Prabhat × UPAS 120 | Andhra Pradesh (AP) | Determinate, early maturity, semi-spreading |
| 27 | GS 1 | Local selection | Karnataka | Indeterminate, semi-spreading, medium duration |
| 28 | GT 1 | Selection of Dabhali | CZ | Semi-spreading, indeterminate, mid-long maturity, large seed size |
| 29 | GT 100 | T 15-15 × S 5 | CZ | Determinate, semi-spreading, early, white and bold seed, resistant to FW and SMD |
| 30 | GT 101 | BWR 24 × Pusa Sweta | Gujarat | Indeterminate, early, spreading |
| 31 | ICP 7035 | Local selection from Bedaghat, Madhya Pradesh | SZ | Indeterminate, medium duration, resistant to SMD, brown and mottled seed, vegetable type |
| 32 | ICP 89049 | C 11 × ICP 8863 | AP | Indeterminate, semi-spreading, medium maturity, brown seeded |
| 33 | ICPA 2089 | ICPL 84045 × ICPL 85037 | AP | Indeterminate, early maturity, brown seeded |
| 34 | ICPL 11255 | MN 8 × ICPL 85010 | - | Determinate, super-early maturity, brown seeded |
| 35 | ICPL 20340 | MN 5 × ICPL 85010 | AP | Determinate, super early maturity, brown seeded |
| 36 | ICPL 7124 | EC 109873 | - | Determinate, early maturity, brown seed |
| 37 | ICPL 7148 | EC 109897 |  | Determinate, early maturity, brown seed |
| 38 | ICPL 84023 | Comp.IoDT-7 × 74092-DTB-15-1-IDT1-B*-13 | A P | Determinate, early maturity, brown seed |
| 39 | ICPL 87154 | ICPL 87 × FSL-1 | A P | Determinate, early maturity, brown seed |
| 40 | ICPL 88034 | ICPL 81 × ICPL 151 | A P | Indeterminate, early-maturity, brown seed |
| 41 | ICPL 91045 | 80576 × Pant-A3-P3* | AP | Indeterminate, early-maturity, brown seeded |
| 42 | IPA 15F | PBJ × SSC-2/33 | UP | Indeterminate, semi-spreading, late maturing, resistant to SMD |
| 43 | IPA 16F | Selection from germplasm line JKH/SSC-3/18 | UP | Indeterminate, semi-spreading, late maturing, resistant to FW and SMD |
| 44 | IPA 2012-1 | Local selection from Allahabad, Uttar Pradesh | UP | Indeterminate, semi-spreading, late maturing, resistant to SMD |
| 45 | IPA 203 | Bahar ×AC314×AC314 | NEPZ | Resistant to SMD, tolerant to Phytophthora Blight (PB) and Fusarium wilt (FW) |
| 46 | IPA 8F | ICP 13673 | UP | Indeterminate, semi-spreading, late maturing, resistant to FW and SMD |
| 47 | IPA 9F | Selection from germplasm line VKG 14/151 | UP | Indeterminate, semi-spreading, late maturing, resistant to FW |
| 48 | JA 4 | No 148 × C 11 | Madhya Pradesh (MP) | Indeterminate, spreading, mid-long maturity |
| 49 | Jagriti (ICPL 151) | ICP 6997 × Prabhat | SZ | Determinate, semi-spreading, early, medium bold seed |
| 50 | JBP 13 | Local selection from Jabalpur, Madhya Pradesh | MP | Indeterminate, semi-spreading, medium maturing, resistant to SMD and tolerant to FW |
| 51 | JKM 189 | ICPL 87119 × P 142 | CZ | Tolerance to drought, moderately resistant to FW and SMD, and Phytophthora stem blight |
| 52 | JKM 7 | ICP 8863 × LRG 30 | CZ | Indeterminate, semi-spreading, mid-long duration, tolerant to FW and pod borer |
| 53 | KPL 43 | Selection from variety Bahar | UP | Indeterminate, semi-spreading, late-maturity, resistant to FW, SMD and tolerant to PB |
| 54 | KPL 44 | Selection of Bahar | UP | Indeterminate, semi spreading, resistant to FW, SMD and tolerant to PB |
| 55 | Kudarat | Local Selection from Sonbhadra district of Uttar Pradesh | UP | Indeterminate, erect and compact, bold seeded, resistant to SMD |
| 56 | Laxmi (ICPL 85063) | (T 21 × BDN 1) × JA 275 | SZ | Indeterminate, semi-spreading, -long duration, suitable for rabi planting |
| 57 | LRG 30 | Local selection of Lam, Andhra Pradesh | A P | White and large seeds |
| 58 | LRG 38 | C 11 × ICP 7035 | AP | Indeterminate, medium maturity, brown and bold seeds |
| 59 | MA 3 | Selection from MA 2 | CZ | Spreading, constricted pod, mid-long duration, resistant to pod fly |
| 60 | MA 6 | MA 2 × Bahar | NEPZ | Indeterminate, spreading, long duration, resistant to SMD and FW |
| 61 | MAL 13 | (MA 2 × MA 166) × Bahar | NEPZ | Spreading, indeterminate |
| 62 | Manak | T 21 × UPAS 120 | Haryana | Indeterminate, semi-spreading, small seeded, early maturity |
| 63 | Maruti (ICPL 8863) | Selection | SZ | Indeterminate, semi-spreading, medium duration, resistant to FW |
| 64 | MN 5 | - | - | Determinate, extra early maturity, brown seed |
| 65 | MN 8 | - | - | Determinate, extra-early maturity, white seeded |
| 66 | NDA 1 | Sel. from Faizabad, UP | UP | Compact, long duration, indeterminate |
| 67 | NDA 2 (NDA 98-1) | Selection from the genotype NDA 1 | NEPZ | Indeterminate, compact, long duration, resistant to FW |
| 68 | Paras | EE 76 × UPAS 120 | Haryana | Indeterminate, semi-spreading, early maturity, brown seed |
| 69 | PAU 881 | H 89-5 × ICPL 85024 | Punjab | Indeterminate, early maturing |
| 70 | PI 397430 |  |  | Resistant to FW |
| 71 | Prabhat | T 1 × T 190 | North Western Plain Zone (NWPZ) | Determinate, extra-early maturity, dwarf plant type |
| 72 | Pragati (ICPL 87) | JA 277 × T 21 | SZ | Determinate, spreading, early, brown seed |
| 73 | PT 221 | Local selection | Maharashtra | Indeterminate, semi-spreading |
| 74 | Pusa 2001 | Np. 148 × UPAS 120 | Delhi | Indeterminate |
| 75 | Pusa 2002-2 | Sel. 90310 × H 88-45 | NWPZ | Early maturing |
| 76 | Pusa 33 | UPAS 120 × C 11 | NWPZ | Indeterminate, semi-spreading, early maturity |
| 77 | Pusa 84 | Pusa Ageti × T 21 | NWPZ | Semi-spreading, determinate, early maturity, brown seed |
| 78 | Pusa 855 | Selection of T 21 | NWPZ | Indeterminate, medium bold seed |
| 79 | Pusa 9 | UPAS 120 × 3673 | NEPZ | Indeterminate, resistant to SMD and Alternaria blight, long duration, suitable for pre-rabi planting |
| 80 | Pusa 992 | Selection from 90306 | NWPZ | Indeterminate, semi-spreading, early maturity |
| 81 | Sharad (DA 11) | Bahar × WR 15 | NEPZ | Indeterminate, compact, white seed, resistant to Alternaria blight and SMD |
| 82 | T 15-15 | Selection from landrace | CZ | Indeterminate, white seed, suitable for vegetable purpose also |
| 83 | TAT 10 | T 8 × T 2 | CZ | Indeterminate, extra-early maturity |
| 84 | TS 3 | ICP 87051 × PT 221 | Karnatka | Compact, indeterminate, white and bold seed, mid-long duration, resistant to FW |
| 85 | TTB 7 | Selection | SZ | Indeterminate, compact, brown seed |
| 86 | TV 1 | Selection of T 21 | Maharashtra | Indeterminate, semi-spreading, medium duration |
| 87 | Type 7 | Selection | NEPZ | Compact plant, long duration, bold seed |
| 88 | UPAS 120 | Sel. of P 4768 | NEPZ | Indeterminate, semi-spreading, early-maturing |
| 89 | Vamban 1 | (Prabhat × Hy 3A) × (T 21 × 102) | Tamil Nadu | Determinate, early, suitable for intercropping with peanut |
| 90 | Vipula | ICPL 151 × Prabhat DT | CZ | Resistant to FW, tolerant to SMD |
| 91 | VL Arhar 1 (ICPL 88039) | ICP 6 × Pant A2 | Uttarakhand | Indeterminate, spreading, early maturity, medium sized seed |
| 92 | WRG 27 | Local Selection | AP | Indeterminate, medium maturity, bold seed, moderately resistant to SMD, tolerant to *Helicoverpa* |
| 93 | WRG 53 | ICPL 332 × ICPL 85063 | AP | Indeterminate, semi-spreading, medium (160-170 days), medium seed, tolerant to Helicoverpa pod borer |
| 94 | WRP 1 | GSI × ICP 8863 | Karnataka | Indeterminate, mid-long duration, spreading |
