# Supplementary material for: New Hypervariable SSR Markers for Diversity Analysis, Hybrid Purity Testing and Trait Mapping in Pigeonpea [Cajanus cajan (L.) Millspaugh]
Source: Front Plant Sci. 2017 Mar 31;8:377. doi: 10.3389/fpls.2017.00377 (PMC5374739; doi:10.3389/fpls.2017.00377)
Supplement: Supplementary file 2 [file Table2.DOC]

**Supplementary Table 2**. List of pigeonpea genotypes screened for *Fusarium* wilt (variant 2) in the sick field

| **S. No.** | **Genotype** | **^*^Percent wilt incidence averaged over two replications** |
| --- | --- | --- |
| 1 | AK 101 | 28.05 (0.56) |
| 2 | AK 22 | 28.40 (0.56) |
| 3 | AK 250173R | 55.50 (0.84) |
| 4 | AL 15 | 87.05 (1.20) |
| 5 | AL 201 | 78.00 (1.08) |
| 6 | Bahar | 90.95 (1.27) |
| 7 | Banda Palera | 3.95 (0.20) |
| 8 | BDN 2 | 95.45 (1.36) |
| 9 | BRG 2 | 87.65 (1.21) |
| 10 | C 11 | 2.20 (0.15) |
| 11 | CO 5 | 29.15 (0.57) |
| 12 | CO 6 | 93.90 (1.32) |
| 13 | CORG 9701 | 62.75 (0.91) |
| 14 | ICP 7035 | 7.55 (0.28) |
| 15 | ICP 89049 | 2.25 (0.15) |
| 16 | ICPL 11255 | 0.00 (0.00) |
| 17 | ICPL 20340 | 4.55 (0.21) |
| 18 | ICPL 7148 | 72.05 (1.01) |
| 19 | ICPL 84023 | 27.20 (0.55) |
| 20 | ICPL 87154 | 7.10 (0.27) |
| 21 | ICPL 88034 | 21.50 (0.48) |
| 22 | ICPL 91045 | 28.15 (0.56) |
| 23 | IPA 15F | 8.95 (0.30) |
| 24 | IPA 16F | 15.25 (0.40) |
| 25 | IPA 2012-1 | 5.60 (0.24) |
| 26 | IPA 203 | 8.75 (0.30) |
| 27 | IPA 8F | 10.10 (0.32) |
| 28 | IPA 9F | 8.90 (0.30) |
| 29 | JBP 13 | 55.80 (0.84) |
| 30 | JKM 189 | 72.85 (1.02) |
| 31 | MAL 13 | 96.70 (1.39) |
| 32 | Maruti (ICPL 8863) | 24.75 (0.52) |
| 33 | NDA 1 | 14.45 (0.39) |
| 34 | PI 397430 | 55.25 (0.84) |
| 35 | PT 221 | 56.25 (0.85) |
| 36 | Pusa 2001 | 86.95 (1.20) |
| 37 | Pusa 992 | 96.85 (1.39) |
| 38 | WRG 27 | 96.85 (1.39) |
| 39 | WRG 53 | 84.30 (1.16) |
| 40 | WRP 1 | 23.60 (0.51) |

^*^Numerals in the parentheses are the arc sine transformed values of percentages
