# Supplementary material for: New Hypervariable SSR Markers for Diversity Analysis, Hybrid Purity Testing and Trait Mapping in Pigeonpea [Cajanus cajan (L.) Millspaugh]
Source: Front Plant Sci. 2017 Mar 31;8:377. doi: 10.3389/fpls.2017.00377 (PMC5374739; doi:10.3389/fpls.2017.00377)
Supplement: Supplementary file 3 [file Table3.DOC]

**Supplementary Table 3** Details of 421 SSR primers used for validation across eight pigeonpea genotypes and polymorphism status of SSRs across six parental combinations

| **S. No.** | **Marker Name** | **^*^Status** | **Forward primer (5'-3')** | **Reverse primer (5'-3')** | **Reference** | **Polymorphism status within cross combinations** | | | | | |
| --- | --- | --- | --- | --- | --- | --- | --- | --- | --- | --- | --- |
|  |  |  |  |  |  | **Type 7 × ICP 8863** | **PA163 A × AK261322R** | **ICPL 88039A × AK 250189 R** | **ICPA 2089 × AK 250189 R** | **ICPL 88039 A × AK 261322R** | **ICPL 88039A × AK 250173R** |
| 1 | CcGM00023 | M | AAATGTTGATAAGAGAATACCACGG | GCATTCCCTTAATATTAGATGATGGT | Varshney et al. (2012) | M | M | M | M | M | M |
| 2 | CcGM00048 | M | AAGCAACACTACGCCAACTT | TCAAAGCATAAAAGCGGACC | " | NA | M | NA | NA | NA | NA |
| 3 | CcGM00340 | M | CGGGGTCCATTATAAGGTTG | CCCATTTGATCCTGAGATTGA | " | M | M | M | M | M | M |
| 4 | CcGM00360 | P | CTCCAATAGAAAATATCAAATCCAA | TCCAAGATTAAGGAAGTGTCTCA | " | P | M | M | M | M | M |
| 5 | CcGM00931 | P | ATCTGATCCCACCTGGTCAT | TCAACCAAAAACATAAAACTCGT | " | M | P | M | P | M | M |
| 6 | CcGM00936 | M | ATTGAACATGCATTGACCCA | TCATTTATTTTTATCTTTTAACCCGAA | " | M | M | M | M | M | M |
| 7 | CcGM01638 | M | TTGGAAGGACTCCACTCCC | ACTCTTTGGAAGGTGGTTCG | " | M | M | M | M | M | M |
| 8 | CcGM01650 | M | TTTCTTTCTCCTTGGTCCCC | GGGAGTTATGAATAAGCAATGGG | " | M | M | M | M | M | M |
| 9 | CcGM01904 | P | TGCCACATGTGCACTTAATGTAT | AAGAGAAAACCAAACAGCCC | " | M | M | P | M | M | P |
| 10 | CcGM01991 | P | ACGTACAAAATAATTATCCAAAACAA | CAAACCATCAATGCCACAAG | " | P | P | P | M | M | P |
| 11 | CcGM02114 | P | GGATCTTTTTATGCATCCTAATGTTT | GGATGACACAAATGTTAATCAAGC | " | M | M | P | M | M | M |
| 12 | CcGM02259 | M | AGGAAATCCACAATCCACCA | ATTCTTAGTGGGTGGGAGGG | " | NA | NA | M | M | NA | M |
| 13 | CcGM02585 | P | GAAGGGAAGGAGGGACGTAG | CAGAGCACACGCGTAGAAAA | " | M | M | M | P | M | P |
| 14 | CcGM02613 | M | GGGTCAGATTTGGTCCTCAA | CGCTATTTTTCGCTTTCCCT | " | M | M | M | M | M | M |
| 15 | CcGM02658 | M | TGCTGGTTTCTCTGTCTTTTTATG | CCTCTTCCAAAAAGCACCAA | " | NA | NA | M | NA | M | NA |
| 16 | CcGM02713 | M | AAAGAGCTCCATCGCAATTT | GCCATTTGACCAAGTCAACC | " | M | M | M | M | M | M |
| 17 | CcGM02747 | M | AGTTACCCGGTGGGATGTTT | AATTGTTGGAGGGGAAGCTC | " | M | M | M | M | M | M |
| 18 | CcGM02953 | M | ACGAAGCGAAGAAGAAGCTG | GCCACGAAATCTTCCTCAAC | " | M | M | M | M | M | M |
| 19 | CcGM03169 | P | AGTATGGGCTGCAGCAAGTT | GCCAATGGTCATAGGGGATT | " | P | P | P | P | P | M |
| 20 | CcGM03373 | P | CGCCAATAAATGACAAGCAA | TGCATGAGTTATAGTCATTGAACAGA | " | M | M | P | P | M | P |
| 21 | CcGM03496 | M | CAACTTGCTTAGGGGACCAA | CGTTCCAGATTTGCCTTGTT | " | NA | NA | NA | NA | M | NA |
| 22 | CcGM03681 | P | GAATATTTTTCAATACCAGGCTGC | GCGTTTCACATGTTCTCATTG | " | P | M | M | M | M | M |
| 23 | CcGM03809 | P | CATAATTCTGATCCTAAACAATAATCA | AAGTCCGACATTGCAATCAA | " | P | M | M | M | M | M |
| 24 | CcGM03896 | M | TTCTGAACAAATGATGATTCAAAC | ATCGACTCAATCCAACCTGG | " | M | M | M | M | M | M |
| 25 | CcGM03905 | M | TTGTGAACACGCAACTTCTCT | TGCTCCCCTCAATTTTCAAG | " | M | M | M | M | M | M |
| 26 | CcGM03916 | M | TTTTTGAGGGGGAGATATAGAAA | AATAGGTACGTGACATTTACAGAATTT | " | M | M | M | M | M | M |
| 27 | CcGM04313 | M | TTGTGAACACGCAACTTCTCT | TGCTCCCCTCAATTTTCAAG | " | M | M | M | M | M | M |
| 28 | CcGM04728 | P | CAAATTCAAACAAATGGGGG | TCCTCGCAGTCACAGTGAAA | " | M | M | P | M | P | M |
| 29 | CcGM04825 | M | TTCCCATTTTATGGAAATATCTAGG | CAACATGTCAACGAATACAAAGG | " | M | M | M | M | M | M |
| 30 | CcGM04943 | P | TGTTATTAACCTAAAACACAAAACTTG | CCGTTACCGACCTAACCTGA | " | M | M | P | M | P | M |
| 31 | CcGM05059 | M | TGTTTAAAAAGGGCTGGGAT | AACTTTTGATTATAAATTTAGACACGC | " | M | M | M | M | M | M |
| 32 | CcGM05108 | P | CGCTTTAAAGAAGGCCAATG | TCCTGCCAGATTTCTGTTTG | " | P | P | P | M | P | P |
| 33 | CcGM05160 | M | TTTCCATCCCTTCGGTATGA | TTTTCGAGAGGCAACTACCG | " | M | M | M | M | M | M |
| 34 | CcGM05227 | M | TAGGGATCATGGGGAACAAA | AAAAACTCATCAAAATCAAAGTAGACC | " | M | M | M | M | M | M |
| 35 | CcGM05317 | M | GCACTCCGAAACAAAAGTCC | AATGCCTTACATGGCCAAAC | " | M | M | M | M | M | M |
| 36 | CcGM05516 | M | CTCGTTATCCAACCCAATCC | TCATTATCAACTCAAAAACCCAA | " | M | M | M | M | M | M |
| 37 | CcGM05543 | M | TCCTCATCAATCACACCTAACTTT | ATTGAGGATCGATGACCGAA | " | M | M | M | M | M | M |
| 38 | CcGM05571 | M | TTTGGGGGTATAATGATCCA | TTCGTGGCATTCTACCTTGA | " | M | M | M | M | M | M |
| 39 | CcGM05661 | M | TTTCTCATCCGATAAACCCG | GGGGAAATCGTTGAGTTTCA | " | M | M | M | M | M | M |
| 40 | CcGM05979 | M | TCAATAAGTAATGGACCTAAGGCT | CCCACAATCCCTAATTTCAAAC | " | M | M | M | M | M | M |
| 41 | CcGM05999 | M | TGTCAAAAATTGGTGGACGA | TGTTGCACAACAAATCACATATT | " | M | M | M | M | M | M |
| 42 | CcGM06000 | M | TGTGGTGTGAATAGAAGTCCAA | TGATGAAGTGATGTCAGAAAGAGA | " | M | M | M | M | M | M |
| 43 | CcGM06013 | M | AAACAGCGTCCGGAGAAAAT | CATCGTTACCGCATTATCACA | " | M | M | M | M | M | M |
| 44 | CcGM06350 | P | AAGCTAACCAAACTTTAGAAACCAA | CATCTAATCAATTCTTTCATCATCA | " | P | P | P | P | P | P |
| 45 | CcGM06356 | P | ACCGTGAGGATATGAGGTCC | TGAAATTCACATGATTTTAACCCA | " | P | M | P | M | P | P |
| 46 | CcGM06568 | P | TGGGCTGGCTTCTCTTCATA | TGAAATTTTGAATGTCTCACCAA | " | M | M | M | M | M | P |
| 47 | CcGM06586 | P | AGAATGTATAAATGTTTGAGTTCACAG | TGTTCAAACACACCTGAATCATAA | " | P | NA | P | M | P | NA |
| 48 | CcGM06587 | P | AGCGGTGCTGTTAACAAAGTT | AAACGACAAAAGGTGGGTTG | " | P | M | P | M | P | M |
| 49 | CcGM06687 | M | GAGACACAAAATGCTTCTACTTCA | GTGCGAGCACTTGTTAGGTG | " | M | M | M | M | M | M |
| 50 | CcGM06954 | M | CGCATTGATTTCGGATCTTT | TCACCAACCTCAAATCACCA | " | M | M | M | M | M | M |
| 51 | CcGM06981 | M | TGATTTCAAGGTATAATTACGGTCAA | TTTGGATTTACACCTGGTGAC | " | M | M | M | M | M | M |
| 52 | CcGM07015 | M | CCACCATTGGACACGGTATT | TGTAAAACATGAGTTATTGAACGGA | " | M | M | M | M | M | M |
| 53 | CcGM07177 | M | TGTGAAAATGGTTGTTTCAAAT | TACAACCCCAACTCCCTCAC | " | M | M | M | M | M | M |
| 54 | CcGM07366 | M | TCCTTAAATCTTAGTTGTGATTCCC | CGCGTAGTACCTTCAGAGCC | " | M | M | M | M | M | M |
| 55 | CcGM07409 | M | GAAAATGACATTGGACATAACCC | TTTTATTTTTCAATCGAGGACG | " | M | M | M | M | M | M |
| 56 | CcGM07675 | P | AATAGTGAGAGTTGGAATAGTATGCAC | TCATCCCTACAACTCATTCATCA | " | NA | M | P | NA | P | P |
| 57 | CcGM07758 | M | GACTCGATGAGTTTGAATTGGA | TGCGATAAATCCAAAAACACA | " | M | M | M | M | M | M |
| 58 | CcGM07858 | M | CATTGCATACCGTAATCATGC | AAAGTGTTGTTGTGGACGCC | " | M | M | M | M | M | M |
| 59 | CcGM07873 | P | GCATGAACGCACACCTTTTA | TCCTGTGCAAAATCTTAAACGA | " | M | M | P | P | P | P |
| 60 | CcGM07918 | M | GCTCCATTTTTAATCATTTTATTTGA | CTGGTCTTTGATCGGTTGGT | " | M | M | M | M | M | M |
| 61 | CcGM07924 | M | TCACTAGCTAAGATCAATGTAGCG | AACGGAAAGGAAAGAGAAAAA | " | M | M | M | M | M | M |
| 62 | CcGM08129 | P | TCGATATGATTTGCACACCC | TTTGCATCCGAAAAAGATCA | " | NA | P | M | M | NA | M |
| 63 | CcGM08278 | M | TGCAGCTAACAGTCACATCAA | GATGTGTCAAACATGATATGAAAATAA | " | M | M | M | M | M | M |
| 64 | CcGM08330 | M | TGCGGTGTTTGGTATAACATTT | GGCAGGGTTTAGCTTAAAAGG | " | M | M | M | M | M | M |
| 65 | CcGM08356 | M | CGTCACACTGTTCAAGTCGG | AAAGTGGAGGGAATGAAGCC | " | M | M | M | M | M | M |
| 66 | CcGM08534 | M | TGGGTTTGTGGTAGGTTGGT | CACTCTGTGGCGGTAAAGGT | " | M | M | M | M | M | M |
| 67 | CcGM08535 | M | TGTACGATACATGGTTAAATTACAAAA | AAGCACGTCATTTGCACTAAAA | " | M | M | M | M | M | M |
| 68 | CcGM08658 | M | TCATGCATACGTGAGAACAAAA | CGACTTTGCCGAATATCCAT | " | M | M | M | M | M | M |
| 69 | CcGM08668 | P | TGGCCTTATTCTACTACCAATACA | GGATTCGATACTAGTTTCTTGCTG | " | P | M | M | M | M | M |
| 70 | CcGM08701 | P | GCATTATTGATTCATCATTTTCG | AAACTATGAGGTGTGATGTGATGA | " | P | P | P | P | P | P |
| 71 | CcGM08736 | M | CGGATTCTTTCGATGTTTGG | AATCAAGAGTGATAATGAGTATTGTCC | " | M | M | M | M | M | M |
| 72 | CcGM08896 | P | AAGATGTTGGAATTGTCGCA | GCCAATTGTAAAAGACTAAACTACCC | " | P | M | P | NA | NA | M |
| 73 | CcGM09142 | M | AAAAATGCGCACTATTCTCTCC | GCATACTACCCAACTTTCCACT | " | M | M | M | M | M | M |
| 74 | CcGM09211 | P | TAGAAGGGGAAGGAAGGACG | AACTTCCCTCAACTCCCGAT | " | P | P | M | M | P | M |
| 75 | CcGM09269 | M | CCAAATTGATCGACACCTCA | AGGATAAAAGCGGGTGACCT | " | M | M | M | M | M | M |
| 76 | CcGM09281 | M | TGCAATGACAGTGGTGACAA | GGTCATTAAAATTCAAAGTAGACCA | " | M | M | M | M | M | M |
| 77 | CcGM09327 | M | AGAGGGTGTATCATGTGCCC | TGATATGTACAGTGAGGGTACGAAA | " | M | M | M | M | M | M |
| 78 | CcGM09344 | M | TCTGACTTTGGCATTTGCAC | TCAAGATTTTGGATTTGAGTATTGA | " | M | M | M | M | M | M |
| 79 | CcGM09431 | M | CATTCGAAATGTTTAGTCTTTGG | TGTTATGGACTCAAGTAACCAACAT | " | M | M | M | M | M | M |
| 80 | CcGM09457 | P | AGTTACGTTGCGTTCTCTTGA | CATCAATGGTATCGACGGTG | " | P | M | P | M | P | P |
| 81 | CcGM09571 | P | TGCAATTCCTTTAACCCATCA | TTTTTACCTGTGACTCAACTCAAT | " | P | M | M | M | M | M |
| 82 | CcGM09707 | P | TTGCATTAGCCAAGCAAGGT | GTTTTAGGGGAAAATGCGGT | " | M | M | M | M | M | M |
| 83 | CcGM09743 | M | ACATGCATGCTTTTGCCTTT | GTGCATTTTTGCAGCCTTTT | " | M | M | M | M | M | M |
| 84 | CcGM09765 | M | TTTTCATCCCAGCTTTTTCC | TTCGGAAAGCTGTAGCAATG | " | M | M | M | M | M | M |
| 85 | CcGM09928 | M | CAAGAAGAAGAGGGAGAAGAAGG | TTCTTATTTTTGTTATTGTTCGTTTC | " | M | M | M | M | M | M |
| 86 | CcGM09960 | M | CCTCCCATCATTGTGGAAAC | TGAAAATCTGCGATGAGTGG | " | M | M | M | M | M | M |
| 87 | CcGM10082 | M | TTTTACCCGTGTCGAAGGAA | AACCCAAGAGCCTCTATGTGAC | " | M | M | M | M | M | M |
| 88 | CcGM10180 | M | CGCCATTTTTATTCTTTTGGG | TCACTGCAATAGACAATTTGATG | " | M | M | M | M | M | M |
| 89 | CcGM10281 | M | CATGTTAAAAAGGGGATTATTTATGG | GGACCACACCTTATAACAACCA | " | M | M | M | M | M | M |
| 90 | CcGM10317 | M | TGAGGGAGAATGTAGCTCCTCT | AAGGACACCTCCCATGTTTG | " | M | M | M | M | M | M |
| 91 | CcGM10349 | M | AGCTCATCCAAAGTTCGAAAA | TTTCAAACAAAACATCAATGTTAAA | " | M | M | M | M | M | M |
| 92 | CcGM10426 | M | AGGGGAGTGGGTTTCAGAGT | CCTCACGAGATTCAAGCACA | " | M | M | M | M | M | M |
| 93 | CcGM10737 | P | TTCCTACTTCGTGGAACCCA | TTCATGTGCAGAATCATCATCA | " | M | M | P | M | M | P |
| 94 | CcGM10754 | M | AAAAACAGTTCCAACAAAATTCAA | GCATAAGTTTGACCGTAAAGAAA | " | M | M | M | M | M | M |
| 95 | CcGM10832 | P | TTCAAAATAAACAATGTTGAACAAA | TCCACGATGATAACTATGATGAATG | " | M | P | P | P | M | M |
| 96 | CcGM10922 | P | GACCACCTTGTACACCGTCC | AGAGGGATACCAATTTGGCG | " | M | M | P | P | P | M |
| 97 | CcGM10927 | M | TTCTGTAACACTCTACACTTTCACAAT | ACCTTCCTCCAATCCGTTCT | " | M | M | M | M | M | M |
| 98 | CcGM10934 | M | AGGCTGCCTCTGTTTTCTCA | CACCTCATCAACCCATCTGA | " | M | M | M | M | M | M |
| 99 | CcGM11045 | M | TCAGCTCATCACTTACAAGGTCA | AACGGGCCTAATCTTATTGTCA | " | M | M | M | M | M | M |
| 100 | CcGM11067 | M | AATCTGGATGATGCATGTGG | CGATGACATAAGTTTTTCTCGTG | " | M | M | M | M | M | M |
| 101 | CcGM11103 | M | TGCTCTTTTGGTACGGCTCT | TGTGGTTTTTCTTTCAGGGG | " | NA | M | M | M | M | M |
| 102 | CcGM11452 | M | TCAAGATAAGTCCAACCAATGA | TTGTCGTGCTCGTATGAGTAGTT | " | NA | NA | NA | NA | M | NA |
| 103 | CcGM11484 | M | ACGTTTCGAGAAAAGGCTGA | TTTCTTTTGGATTTCGCTTCA | " | M | M | M | M | M | M |
| 104 | CcGM11515 | M | CAACGGATCCCTCCTTGTAA | GAATGCAAGAACACTGAGCG | " | M | M | M | M | M | M |
| 105 | CcGM11519 | M | CGTAACACCACATTCATCACCT | TCCCCACTCTTTTAAATTTCG | " | M | M | M | M | M | M |
| 106 | CcGM11620 | P | AACTCCAAAATCCAAAACTCG | CCTATGATCCAACTCGACCC | " | M | P | M | M | P | P |
| 107 | CcGM11658 | P | CATTATTCAACCCCTTTTAGTGTC | TCAAATTTGGAAGTCGTTGAGA | " | NA | NA | P | NA | P | NA |
| 108 | CcGM11877 | M | TGTATAAATAAAGCTCTGTACCCAAAA | CCGTGAGCTATTGGTCTGGT | " | NA | M | NA | NA | NA | NA |
| 109 | CcGM12037 | P | CAAACACTCCCCATTATATATTCGTT | AAGTCATTCCTTGACTTGAATTTTT | " | M | M | P | M | M | P |
| 110 | CcGM12038 | M | CGCGGTTTTTAACTTCCAGA | CTTCCCTACTTCCCCAAAGG | " | M | M | M | M | M | M |
| 111 | CcGM12067 | M | GACACCTCTTCCCCCTCTTC | AACCGCAGTAGCAGTCACCT | " | M | M | M | M | M | M |
| 112 | CcGM12109 | M | GTGCTCACCCCTGGTCATAG | TGAATCACCATTTTGTGTGGA | " | M | M | M | M | M | M |
| 113 | CcGM12217 | P | GCGAATTGGCTGGGTTAATA | TGTAATGCACAGGTAGACGCA | " | M | M | P | M | M | P |
| 114 | CcGM12275 | P | CTTGGTGAGCCTGGGTTTAG | ACATTCCACCCAAAACATCC | " | M | M | P | M | P | M |
| 115 | CcGM12371 | P | AAGGTTAAAGGTGAATGGGGA | TGGCTTGACATGCAAAGAAT | " | M | M | P | M | P | P |
| 116 | CcGM12576 | P | ACGTGGGTCAAGGACAAATC | TCCCTGGCACTAACAAAACA | " | M | M | P | M | M | P |
| 117 | CcGM12626 | M | TCAACCTAAAACACAAAACTCGTC | CGAAAAGCCTGTGAAACGA | " | NA | M | NA | NA | NA | NA |
| 118 | CcGM12662 | P | TGAGCAAATGAAGTCAGGAGG | CACCGTTGTAAACAGTCCCA | " | M | M | M | P | M | M |
| 119 | CcGM12694 | P | TAAAGATGCATTCCAACCCC | AAAACAAAATGCTTTTAATCGTAAAGA | " | M | M | P | P | P | M |
| 120 | CcGM12712 | M | TTTGGTTCCAATATTTTGAAGG | AAAACCAAGCTGCAAACATTA | " | M | M | M | M | M | M |
| 121 | CcGM12764 | M | GAGATACGTATTACCTGTCACTCACT | CCTGGAGAGTAGTTTGGGCA | " | M | M | M | M | M | M |
| 122 | CcGM12994 | M | AACGGAAGGAGGGACGTAGT | AGATGAAGCACGAGCGAAAT | " | M | M | M | M | M | M |
| 123 | CcGM13014 | M | GAACCTTCAATCTATAACCATGAAA | GGTTGCATATAGTTTGACTCGC | " | M | M | M | M | M | M |
| 124 | CcGM13040 | M | TGCAGTGAATGCAATATTTTAGA | CAATAAGAAAAAGCTAATCACTCTCA | " | M | M | M | M | M | M |
| 125 | CcGM13099 | M | TTGAATTGAGTGATGATATAGTCGG | AAACAAGCAAGTGCATATTAACAAA | " | M | M | M | M | M | M |
| 126 | CcGM13163 | M | AAGGAAAAAGTGAAAGTTTTGTCA | CCTTTGTATTAACTTCCCAACATGA | " | M | M | M | M | M | M |
| 127 | CcGM13187 | M | CGTGCAAAATATATTGGAGATTCTT | CCACAGAGCTCAAAGGCAA | " | M | M | NA | M | NA | NA |
| 128 | CcGM13188 | M | TCATGGTGGAATGACACTGG | TGGCACCAAATTAACCATCA | " | M | M | M | M | M | M |
| 129 | CcGM13213 | P | CCATGCTATCAACCTAAAATACAAAA | CCCGTTAACCAACTCAATCTG | " | P | M | M | M | P | P |
| 130 | CcGM13254 | P | AGGCTTTTGTACCACCGTGT | CATGTATGTTCCCTGTATTTAATTTG | " | M | M | M | P | M | M |
| 131 | CcGM13268 | M | GAGAACCCTAGAGAGGGAAGC | GGGATGGACTCCAAGCTAGA | " | M | M | M | M | M | M |
| 132 | CcGM13288 | P | CAAGTTGTTTCAAACTTTCCTTCA | GGGACCAAATTGACCCCTAA | " | M | M | M | P | P | P |
| 133 | CcGM13289 | M | CCCAATTTTTGTATCCGATCT | GGGTGTCGCAAAGAGTTGAT | " | M | M | M | M | M | M |
| 134 | CcGM13405 | M | GAGGACTCCTGGTCCTAGCC | CCGATGAGAGGTTGGTTCAT | " | M | M | M | M | M | M |
| 135 | CcGM13428 | P | GGAAGAACAGTTTTGGAAAGAA | CACACGCAAAGTTGATGGAC | " | M | M | M | P | M | M |
| 136 | CcGM13439 | M | TCATTATCTTTATTATCGTCATTGTCA | AACAACATTGGGTTGCATTG | " | M | M | M | M | M | M |
| 137 | CcGM13503 | P | GAGATAGGAAGAAATTGCCGAA | TGAACTCACTTTAGAATTTGTGTGTG | " | M | P | M | M | M | M |
| 138 | CcGM13505 | P | AATCGATGTCGAGCGAGAGT | ACAACAGAACAAGTGCCACG | " | P | M | M | M | M | M |
| 139 | CcGM13512 | M | GGCTCAAAACAATTAAGCCAA | CAAATGGGTCGAGATGGATT | " | M | M | M | M | M | M |
| 140 | CcGM13537 | P | AACATGAGTTATTAAATAGAGGTAGCG | CATGATAAAAACTTGATAAACAGGACA | " | P | M | M | M | M | M |
| 141 | CcGM13637 | P | AGGTTGATTCATGAAAATTGGA | TGCCAATACCTATGCTTCCTTT | " | M | M | M | P | M | M |
| 142 | CcGM13650 | M | GCATATAAGGAGAAAGTAAATTTTTGA | CATACCCCCAATATCCCATTT | " | M | M | M | M | M | M |
| 143 | CcGM13712 | P | GAGTAGACGCGAACGTGTGA | TTGTCCAATCTTCACGATTCC | " | M | M | M | M | M | M |
| 144 | CcGM13714 | M | TCAGACTCAATCCAACCCAA | TGTTATCAACCTAAAACACAAAACTTG | " | M | M | M | M | M | M |
| 145 | CcGM13722 | M | CTTTTATGATGTTGTTAGAATTTTTCA | AAACGAACAACTTCCAGAGTATTTTT | " | M | M | M | M | M | M |
| 146 | CcGM13766 | P | CTGGTGCATAAGACACCATGT | GGTGGTGCGTGGCTACTAAT | " | M | M | M | P | M | M |
| 147 | CcGM13823 | M | GGAAGGACGTGATGGTCATT | GCAGAATCTGGTTGGTCTCC | " | M | M | M | M | M | M |
| 148 | CcGM13944 | P | GGCATGACAAAACGAACCTT | AAAGAGTTGCGCCCTGACTA | " | P | M | M | M | M | M |
| 149 | CcGM13964 | P | TGCCCTAGCTATGCCAGATT | TGGACACAGCACACAAGACA | " | M | M | M | P | M | M |
| 150 | CcGM14000 | P | TTTCGAACCCTAATCCGTTTT | TTGGTGTCGGTTTCTTAGGG | " | M | M | M | M | M | M |
| 151 | CcGM14057 | P | CAACTGTTTATTTTAATTTCTGTGTGA | AGATCATATAACAAGTATTTCAAACCC | " | P | M | M | M | M | M |
| 152 | CcGM14064 | P | TTTCAAGGCCAAATCAAACC | AACATAATTCACGTCGCGTTC | " | P | M | M | P | M | M |
| 153 | CcGM14109 | P | TCCATAGGTGGTCAAATGTCTG | CTCCTCACAAATGCATGCTC | " | M | M | M | M | M | M |
| 154 | CcGM14169 | P | CCATTCTACCAACCTAAAACACAA | CCGTTATCCAACTCAATCCG | " | P | M | M | M | M | M |
| 155 | CcGM14207 | P | CAAAATAGGGTAATTTCGTCATCA | AGCTTTGAGCAGAAAACTTGC | " | M | M | M | P | P | P |
| 156 | CcGM14230 | M | ATTCATTCGGAATTGCCTTG | AAAAGAAGCATTGCACACCC | " | NA | NA | M | NA | M | NA |
| 157 | CcGM14251 | P | AAAATTGATACGTGCTTTAACTCAA | TCTGTCAAGTTTCCCTGCACT | " | M | M | M | M | M | M |
| 158 | CcGM14252 | P | TGGTCCCCTCGAAAATAAAA | GGGAATTATATCTTTGCCCCTC | " | P | M | P | P | P | M |
| 159 | CcGM14398 | M | GTTTTCTGTAGGTTGTGCTATTTTT | TCCCTGTCCGCTGTTTTATC | " | M | M | M | M | M | M |
| 160 | CcGM14419 | M | TGAAAACAATTCATTTTGGAACA | GACAAAAGGAATAACATGTTGAGAGA | " | M | M | M | M | M | M |
| 161 | CcGM14447 | P | TGTGTTTTCAACCTGTTACCTGA | TGTTATCAACTTAAAATACAAAACTCG | " | M | M | M | M | M | M |
| 162 | CcGM14463 | P | TCCTTTAGACCTAATCACCCG | GGCCAAGATAACGACCAAAA | " | P | M | P | P | M | P |
| 163 | CcGM14475 | P | AAACCAGTGCTAATGTCGTCAC | TTCCCTCACTTGAATTGAACC | " | M | M | M | P | M | M |
| 164 | CcGM14478 | M | TCTGAATTCAACATATGAATTTTTGT | GAGAAAGAGATACCTTGGGTCTTT | " | M | M | M | M | M | M |
| 165 | CcGM14521 | P | TGTTAGTGACCCGACCCAAC | TCAACTCAAAAAGCCAACATT | " | P | M | P | P | M | P |
| 166 | CcGM14561 | P | GGTTTGTCATAACCAAAACTCCA | GACGACAATGGTTACAGCCC | " | M | P | M | M | M | M |
| 167 | CcGM14572 | NA | ATTTGCGAAAGGGATGAAAT | TGCTTCATTCAATTTTCGATT | " | NA | NA | NA | NA | NA | NA |
| 168 | CcGM14603 | M | CAATTTTCACAACTTTATCAACACA | AGTCCTTGAGTGGCGTTGAG | " | M | M | M | M | M | M |
| 169 | CcGM14613 | P | TTACTGTTGCTGCTGCTGCT | AGCACGCTGTGATCATGAAG | " | M | M | M | M | M | M |
| 170 | CcGM14616 | NA | GCTTGTAGCATCTCTTGCCC | CATGGTGAATTGCTTGAGGA | " | NA | NA | NA | NA | NA | NA |
| 171 | CcGM14623 | M | AAGTTGCTTTGTTCCCCCTT | TTTGTCTATTTCTTTGCGGTG | " | NA | M | M | M | M | M |
| 172 | CcGM14720 | P | CAACCTGATCAAAACCCGAT | AAGAGTGACGGTGAGTAATGTCTT | " | P | M | M | M | M | M |
| 173 | CcGM14753 | P | TGTGTCCGCTTAATGAACTTG | TATGCTTCAGCGCAAGACAA | " | P | M | M | M | M | M |
| 174 | CcGM14772 | P | GGTAATATTTATTGAGAATCCACACTT | TTGGGACATGGGAGTTTCAT | " | M | M | M | M | M | M |
| 175 | CcGM14774 | M | TTTGGTGATGCTGGTTTTGA | ATGCCTGAAAATGCTTCCAC | " | M | M | M | M | M | M |
| 176 | CcGM14845 | M | GAGGATACACCAATAATCTTCTCG | AAACATAGTGTAAATGAAAAGAAAACG | " | M | M | M | M | M | M |
| 177 | CcGM14907 | M | CAAAAATTTGCAAGAAAAGTCG | TCATTTTTCATTTTTCTTTTTCATC | " | M | M | M | M | M | M |
| 178 | CcGM14937 | P | TGATAAATGTCCACCTCATTTTT | CGTCTAAACTTAATCCAACTCAACC | " | M | M | M | M | M | M |
| 179 | CcGM14953 | P | TTGCTTTTTGATGTGTAGGCA | TTTCGATTTAACGGTTCGATTT | " | M | M | P | M | P | P |
| 180 | CcGM14962 | P | GACGCTTGCGAGAAGACTTT | GCAATCGAGTTAATCATGAAGTG | " | M | M | M | M | M | M |
| 181 | CcGM15117 | P | GTGTTGGGCAACCTGTATCC | TCAATGAGTGTAACATGTCCTCTTC | " | M | M | M | M | M | M |
| 182 | CcGM15126 | M | GGATGAAATTTGCAAAACGA | TTTGTATACATGAAAATGGTGAATC | " | NA | M | NA | NA | NA | NA |
| 183 | CcGM15129 | P | CATTAGCAGAAATATGCATGGAG | GGCTAAGATGAAAAATCCAAACC | " | P | P | P | M | P | M |
| 184 | CcGM15165 | P | CACAACGCCAAAGCTGTATG | CCGGAAGACCAAACTCATCT | " | P | M | M | M | M | M |
| 185 | CcGM15219 | M | AACTCAACTCTAGAACGGCCC | TAGGCATGGCAATCTAACCC | " | M | M | M | M | M | M |
| 186 | CcGM15232 | P | CACAGTGAATATTGTCCTGAGCA | AACCCGTTACTAACCCGACC | " | P | P | M | M | M | M |
| 187 | CcGM15325 | P | AGAGCATCTTGAGTTATTCGGT | CCTGTGCTCGAATTTCTTCC | " | M | M | M | M | P | M |
| 188 | CcGM15339 | M | TCCCCTTTACTGTTACCGCTT | AAGGGGCGTTCTAGGAAAAA | " | M | M | M | M | M | M |
| 189 | CcGM15424 | M | TCGAGCAAGGGTGAGAAAGT | GGCTTATTCGACTTGAACAAAA | " | M | M | M | M | M | M |
| 190 | CcGM15449 | P | TGTTGACATTCATTTACTCCCG | ACGGGGTCTCACAAAAACAA | " | P | M | P | M | M | M |
| 191 | CcGM15473 | P | CGAATAAACTGCATTCGTAAGCTA | TCACGTGACAAACACACAATTC | " | M | P | M | M | M | M |
| 192 | CcGM15508 | P | TGGTGATGCCAATGTCTTCT | TGCAACAAGGTTGAGCTGAG | " | P | M | M | P | M | M |
| 193 | CcGM15605 | P | ATTGGTGGAGTTTGCATAAAA | AACATGTCACTTCCCTTAGTAAAAA | " | P | P | M | M | M | M |
| 194 | CcGM15606 | M | TGGAGAGGGAGAAAGGTCAA | CCTATTGTCTTGACACGTTTGG | " | M | NA | NA | NA | NA | NA |
| 195 | CcGM15638 | M | TCCTTCCCTCTTCTCATTTCC | TGCATTCTTATTCACCTGTGG | " | M | M | M | M | M | M |
| 196 | CcGM15710 | P | TGTTGACATTCATTTACTCCCG | ACGGGGTCTCACAAAAACAA | " | P | M | M | M | M | M |
| 197 | CcGM15748 | M | TGCGGGTGTAGATATTGGGT | TATGGATGCTTGACAGCAGG | " | M | M | M | M | M | M |
| 198 | CcGM15803 | P | TGAAATTGAGTTTTAGGTGTTTCA | TTCATTTCTCCCGCAAAATC | " | M | M | M | P | M | M |
| 199 | CcGM15859 | M | TTCATTTCTCCCGCAAAATC | TGAAATTGAGTTTTAGGTGTTTCA | " | M | M | M | M | M | M |
| 200 | CcGM15990 | M | CCAACAAACATTTAATGCAAGA | GCAAGTAAATGAAGTTAGGAACACC | " | M | M | M | M | M | M |
| 201 | CcGM15998 | NA | TTTAGAATTAACGACAAAACCCTTC | CAACACCTCTCAACTTATAACTCTCAA | " | NA | NA | NA | NA | NA | NA |
| 202 | CcGM16001 | P | TCGTAACTTTCCTCCTCGACA | GTTCTTCTGCCATTCCCTCA | " | M | M | M | P | M | M |
| 203 | CcGM16048 | P | ATGGAGAATCGTCGCTCATC | TTTCACCTGATGGAGATTTGC | " | P | M | M | P | M | M |
| 204 | CcGM16285 | P | TCCACATCACCACAATCACA | CACTGTCACCACCGTTTTCA | " | P | M | M | M | M | M |
| 205 | CcGM16291 | M | AAGGCACGAGGTGTCTCAAC | TCCAATTCAATCCGATCAAAA | " | M | M | M | M | M | M |
| 206 | CcGM16303 | P | CAGTGTTCGGCTCAAACTCA | CCCGATCAAATAATCGTTCAA | " | P | M | P | P | M | P |
| 207 | CcGM16323 | P | GGGAAGTGAAAATTCTCGTTCTA | TCACACCTTTAATTTATGTCCTGAA | " | P | M | P | M | M | P |
| 208 | CcGM16417 | P | AACATGGGAAACAACTAAGATGG | CCGCTATGTACAGTGCTTCA | " | M | M | M | M | P | M |
| 209 | CcGM16497 | M | GTTCTGGAAGCAGAACTGGC | ATCAGCCAAGCAAGGTGAAT | " | M | M | M | M | M | M |
| 210 | CcGM16506 | NA | AAGGTTTCAGGGGAAAATGC | ATCAGCCAAGCAAGGTGAAT | " | NA | NA | NA | NA | NA | NA |
| 211 | CcGM16529 | P | TGATGATGCAAACCCTTGTG | AAGGATGGATTATGTAACGACC | " | M | P | P | M | M | M |
| 212 | CcGM16545 | P | TGTTATCAACCTAAAACACAAAACTT | ACCCATCCATTATTGACCCA | " | P | M | P | M | P | P |
| 213 | CcGM16546 | P | TTGTCGCATTCCTAGCATTC | TCTTCTAAACATGCACCTATAAACTGA | " | M | M | M | M | P | M |
| 214 | CcGM16584 | P | TGCACAACAAATCACTCTCAAA | AAATATTGGCAGCTGGGTTG | " | P | M | P | P | M | P |
| 215 | CcGM16606 | NA | TGAAATTTGATGACTAATTTGGG | TCGCCTTTCTTGCTTTTCTC | " | NA | NA | NA | NA | NA | NA |
| 216 | CcGM16612 | P | AAAGATATGATTGGTTGCTATAAGAGA | TGCAATATGCGTGGGTAAAA | " | M | M | M | M | M | M |
| 217 | CcGM16633 | P | CAAAACATGGAATGGGAAATG | GATCAATGCCATTTTGAGGG | " | M | P | P | M | P | P |
| 218 | CcGM16694 | M | TCCATAAATTCAATCCACCGA | GGCACAAAATGTCTCTTTCCC | " | M | M | M | M | M | M |
| 219 | CcGM16705 | NA | AATGGAACATCACAAACCTTTT | GCCAAATGGGAGAATATTGG | " | NA | NA | NA | NA | NA | NA |
| 220 | CcGM16723 | P | CATTTAAAGCGGGAATGTGG | CAATTCACCATTTTACACTCTTTTT | " | P | M | M | M | M | M |
| 221 | CcGM16750 | P | TGCACATCAAAACAAAGAGTGTC | AGAACATTGTTGCAAAGGCA | " | P | M | M | M | M | M |
| 222 | CcGM16757 | NA | GACGGAAGTCCCAAATTGC | TTTGATCGATTATTAAACCAACTTAAA | " | NA | NA | NA | NA | NA | NA |
| 223 | CcGM16772 | P | CCACATGTGAGTGGATCTGAG | TGCAAAATTCAAATTTACTCTCCC | " | M | P | P | P | M | P |
| 224 | CcGM16775 | P | TTCTTTGGGAATTTATACGAACA | CTTTGGGCCTAAGCCTTCAT | " | P | M | M | M | M | M |
| 225 | CcGM16799 | P | AATGTAAAAGGGGAGCCGAT | TTCGTAAATGTCAATCTCAAACG | " | P | M | M | M | M | M |
| 226 | CcGM16802 | P | ATTAGCGCCCAAGAGACGTA | GCCTTCACTGATTATGATGCTT | " | P | M | M | M | M | M |
| 227 | CcGM16808 | NA | GAGAAAAATAAAGATATGGGAGGG | CCTCTCTCCCTCTCCCCC | " | NA | NA | NA | NA | NA | NA |
| 228 | CcGM16858 | P | CCAACAGAAACATGGGGTTT | TGCACGTGATTAGACTTGGG | " | M | M | M | P | M | M |
| 229 | CcGM16887 | P | GACCGTGATCTTGGTTTTTCA | AAGAGAGTGGCGAGTATTAAAAAGA | " | M | M | M | M | M | M |
| 230 | CcGM16948 | M | ATGACGTGCAGATTTGATGC | GTCCCAAAACATGACCAACC | " | M | M | M | M | M | M |
| 231 | CcGM17051 | **P** | CTCTATCGCAAAAGGCAAGG | AAAAGTGAAGACATCGACAAGGA | " | P | P | M | M | P | M |
| 232 | CcGM17100 | P | CCTAATTCAATTGGTTGATGATGA | AAATTGAATGCCTAATTGTGGA | " | P | M | M | M | M | M |
| 233 | CcGM17150 | P | GAAAGATATTTGTTGAAAAAGTTGAA | TTTGCGTAAATCAGATCCACA | " | P | M | P | P | P | P |
| 234 | CcGM17154 | P | TCGCTGACATGTAAGGTGAGA | CAGCTTATTACCTGCCGTCC | " | M | P | M | P | M | M |
| 235 | CcGM17176 | P | AGGAATCTCATCAACGGTGG | CCATCCGTGCGAGATAAGAT | " | M | NA | M | P | M | M |
| 236 | CcGM17341 | M | CGTACAAAATAATTGTCCAAAACA | GTGGGTTTGCCTAGGACGTT | " | M | M | M | M | M | M |
| 237 | CcGM17365 | NA | AGCGATTGAGCGAAAGAAAA | AAAGTTGCATTGAAAATCATGAGA | " | NA | NA | NA | NA | NA | NA |
| 238 | CcGM17379 | P | TTGTGAAATTGAGTTTTAAGCGTT | TTGGTGAAATATCAACACTACGG | " | M | M | M | M | M | M |
| 239 | CcGM17425 | P | TGAGCCATGATCCAACTTGA | ATCATGCGCAGATGGTTACA | " | P | M | M | M | P | M |
| 240 | CcGM17438 | P | GATCCAGAAATTGACAAGCCA | GCATGGAAAGAGGGTTAGGG | " | M | M | M | M | M | M |
| 241 | CcGM17475 | P | TGCAAGCGAATACAACATCA | TGAGAGGCAAAGTGATCTACTAAAAA | " | P | P | P | M | P | P |
| 242 | CcGM17543 | P | AGGTTCCTCCCTGGTATCGT | CCCTTAAATAAGACCTAAAAGGAAAA | " | M | M | M | M | P | M |
| 243 | CcGM17611 | P | AAGCAAGGGTGACAATGAAT | CAACCCAATCCGATCAAAAC | " | P | NA | P | P | P | P |
| 244 | CcGM17614 | P | GCCTGTAGCTCGTAAGAAAGC | TGAGCTATTTGGTAACAACTTTGG | " | P | M | M | M | M | M |
| 245 | CcGM17620 | P | GTTCCATCAACCTAAGCCCA | CACTTCCTTTCTCGAACCCA | " | P | M | P | P | P | P |
| 246 | CcGM17648 | P | CTTGTCCCAAGACTGTGCCT | GAAAACTATACAAAAGAGCATACGGA | " | M | M | P | P | M | P |
| 247 | CcGM17657 | P | AACAACAATTTGCAGTTTCGAC | TCCATTAGCGAGGAGCTTGT | " | M | M | M | M | M | P |
| 248 | CcGM17703 | M | ACCTCAACGAGGTCCATCAC | GTCAACCACCATCAGCAATG | " | M | M | M | M | M | M |
| 249 | CcGM17797 | P | CAATGAATATTGTCTTGAACAAATGA | CGATCCAATCCAACCTAATCA | " | P | P | M | M | M | M |
| 250 | CcGM17816 | P | GTGTTTTTGACCCATGACCC | TTGACTCAAACAGCCAACATT | " | M | M | M | M | M | P |
| 251 | CcGM17845 | P | CAATGAATATTGTCTTGAACAAATGA | CCCAACCCGATCAAAATCTA | " | M | P | M | M | M | M |
| 252 | CcGM17946 | P | AAAAATGATTTGTGTACGAGTTTTT | AAATTCGACTTGCTGAAATCAA | " | M | P | M | M | P | M |
| 253 | CcGM17970 | P | GGGGTAAACTTGGTGTGTTGA | AAACTTGTTCTTTTCCAATACACTTT | " | P | M | M | M | M | M |
| 254 | CcGM18008 | P | ACGGCGAAACCTTAAAACAC | TCTCATGCCTAGGCTCAACC | " | M | M | M | M | M | M |
| 255 | CcGM18041 | P | CCTTATGTGAAGCATGGAAGC | TTCACAATTTCATTTTCATTTGC | " | P | M | P | P | M | P |
| 256 | CcGM18042 | P | CACCTTAGTTGGTTTCACCCT | TCACAACTTGCACCTTTAAGC | " | M | M | P | M | M | M |
| 257 | CcGM18069 | M | CCTTCAAGGTTCCCAACATC | CCTCCTTAAGCCACCCTCTC | " | M | M | M | M | M | M |
| 258 | CcGM18135 | NA | CGATTCTTTTATAACCGATCCAA | CAGAGTTTCTCAACAACACTCCTT | " | NA | NA | NA | NA | NA | NA |
| 259 | CcGM18196 | P | TTTTCCAACGCGAGTAATGA | AATACCCAAGTCCGTGATGC | " | P | P | M | P | M | M |
| 260 | CcGM18273 | P | TTTCAAGCTACCGTAGAATTTCTCT | CATCCACCCTGCTTGAATTT | " | M | P | M | M | M | M |
| 261 | CcGM18291 | P | CTCAAGGGTGAGCTGTGTGA | CCAAACGTTGTGAACATATGAA | " | M | M | P | M | M | P |
| 262 | CcGM18384 | P | TTCACATTCCTTGTTCATTTCG | GGTTAGGGATACCCTACCAAACA | " | M | M | M | P | P | M |
| 263 | CcGM18428 | M | CCTCCTTGTTCTTCAGAGCG | TCGTTAAAATTTGATGTAGAACAGGA | " | M | M | M | M | M | M |
| 264 | CcGM18474 | M | GGGGTGATACCTGCAAAAGA | GGATCTCGTCCCAGATCAGA | " | M | M | M | M | M | M |
| 265 | CcGM18517 | P | GAAATGCTTCCCAATCAAGG | TCCATCATTATACATTACATGCTTTT | " | P | M | M | P | M | M |
| 266 | CcGM18521 | M | TGAGGATGATGGTTCAATGG | AGTTGGATTGTCGTGCATTTC | " | M | M | M | M | M | M |
| 267 | CcGM18538 | P | TGAAATCCTGTTCTTGCATCA | GCATGATGTCGCATTCTTGT | " | M | M | M | M | M | M |
| 268 | CcGM18570 | M | TGTCGCATTCCAATAATCCTT | GATTATGAGGCATTTCCTTAATCTT | " | M | M | M | M | M | M |
| 269 | CcGM18590 | M | TTGCTTCAAGACCCAACAAA | AGTGAGTGAGTGAGGATGATGC | " | M | M | M | M | M | M |
| 270 | CcGM18599 | P | TTTTGAAATACGACTTTCACTTTAGAA | GTCTCACTCATCTATTCACAATTTTT | " | M | P | M | M | M | P |
| 271 | CcGM18676 | P | GCCTTTGCTCACGGATACAT | TTCTCAAACAACACGGGTCA | " | P | M | P | M | M | M |
| 272 | CcGM18681 | P | TTGTTGTGTTAATGTTTTGCGA | GAACAGTGATTTGAGGGGGA | " | P | M | P | M | P | P |
| 273 | CcGM18684 | P | TCGTGTTAATGTTTTGCCACA | CAGTGAGATTTGAGGGGGAG | " | M | M | P | M | P | P |
| 274 | CcGM18785 | P | TGTGGTGAGGGACACCAGTA | TCTCCGGTGGCTAAGAAGAA | " | M | M | M | P | P | M |
| 275 | CcGM18806 | M | GCACAAGACCAACAAACGAA | TTTGACACGTGCATGCTTTT | " | M | M | M | M | M | M |
| 276 | CcGM18867 | P | GGCACAAAATGTCTCGATCTC | GGCAATTAACTCCGAACCAG | " | M | M | P | P | P | M |
| 277 | CcGM18871 | M | GCGGTATTCCATAAATGATGTTG | TGTGAAGGAATGTATGGGAGAA | " | M | M | M | M | M | M |
| 278 | CcGM18873 | M | TTCAGCACCTCGAACACAAG | GGAAAAGCATGAAAAGCAGG | " | M | M | M | M | M | M |
| 279 | CcGM18876 | P | TTTCTGCTACCCAGTGCAAA | ATACACCCATTTTCCATGCC | " | P | P | M | P | M | M |
| 280 | CcGM18893 | M | TGGCCATCATTTCATATGCC | CTTCAGAGGAGCTGAATCGG | " | M | M | M | M | M | M |
| 281 | CcGM18923 | P | GAGAGTGGTCAACACAGGCA | CCCAACTAAGGACTCTCCCA | " | M | M | M | M | M | M |
| 282 | CcGM18924 | M | AAGACATGTGATCCAAACACCTT | GGTGATCAACATAAAAGTTGGAGA | " | M | M | M | M | M | M |
| 283 | CcGM19093 | M | ACGGTTAGATTCGGTGTTGG | ATCTCTGCATCACCACCCTC | " | M | M | M | M | M | M |
| 284 | CcGM19108 | P | CCTAGCACAGAAGGGAATGC | TTAGGGGCACAATCAAAAGC | " | M | P | M | M | M | M |
| 285 | CcGM19123 | P | CCAATTGTGATGTGTTCCCA | TTCAACTCCCATCCTACCCA | " | M | M | P | P | P | P |
| 286 | CcGM19136 | P | TCTCCTGGATGTTGGTCTGA | ATTTGGTGCAGGAGACTTGC | " | P | M | M | P | M | M |
| 287 | CcGM19144 | P | TGAAACCAATCCTCATTGCAT | TTTTGAGTTTAATTATGCATCGTGT | " | M | P | M | M | M | M |
| 288 | CcGM19152 | P | CACATATTCACAGCCAAACCA | GGATCTCGAATTTTGATTTCCA | " | P | M | M | M | M | M |
| 289 | CcGM19207 | M | AACGTGGTTTTCTGATTCGG | ATCCACCCACCAAACACACT | " | M | M | M | M | M | M |
| 290 | CcGM19217 | P | GGTCGCAAAATTTGGTTTTC | GCCTCGTTCCTTTCTTACCC | " | M | P | P | P | M | P |
| 291 | CcGM19277 | P | CTATTGCCTAGGTCCCCCTC | AATGAGATCAACGCAAAGGG | " | M | M | M | M | M | M |
| 292 | CcGM19285 | P | CAACAAGTGTATGCCATTCCA | TCAGCTCTAAACTAATCCCCCA | " | M | M | M | M | M | M |
| 293 | CcGM19325 | P | ATGCCCCAACAATGTTTCTT | AAGGGTTTTGCTTCTCAACAA | " | M | M | M | M | P | M |
| 294 | CcGM19413 | P | TCCACACAAAAGCAGTGAAGA | GCCATTTATTTCCACACCAA | " | M | M | M | M | P | P |
| 295 | CcGM19472 | P | CGAATGCATACGTGAGAACAA | TGTGGCTTGAACCTTATTGG | " | P | P | M | M | M | M |
| 296 | CcGM19565 | P | CTCTTCTTGTTGTCCCTCGC | GCAGTTCTGGAATACCTCGC | " | P | M | M | P | M | P |
| 297 | CcGM19566 | P | TGAGCGAGTGAAAAGCAAAA | GGCACTGTTCTTAGGCTTCC | " | P | P | M | M | M | M |
| 298 | CcGM19568 | NA | CTCTTCTTGTTGTCCCTCGC | GCAGTTCTGGAATACCTCGC | " | NA | NA | NA | NA | NA | NA |
| 299 | CcGM19614 | M | TCACTCGCTAAGCAATCAAAAA | GCCACGTTCACGTATTGAAG | " | M | M | M | M | M | M |
| 300 | CcGM19652 | P | TCCCTAATAGCCTAATGGTCCTT | TCCACATGTGATTATGGCTCA | " | P | M | M | M | P | P |
| 301 | CcGM19653 | P | GTGATGCTGAGATATTCTTGTCC | CATTTGAGCTATTCACTTCTTTCAA | " | M | P | M | P | M | M |
| 302 | CcGM19665 | M | CCGACACAGTTTCAAGCGTA | CAATAAGAAGCAACGCCCAT | " | NA | NA | M | NA | M | M |
| 303 | CcGM19670 | M | GAAACGAAAGAGAGGGAGGC | TGATCTATATGGTGTCACATTATTTGC | " | M | M | M | M | M | M |
| 304 | CcGM19705 | P | TGAGAACATATTTGAGATGAGATGAA | TGATAGTGAAATAATGGTGATGACG | " | M | M | M | M | P | M |
| 305 | CcGM19740 | NA | GACAAGGGGAAATTCAAGCA | TCTCTCTTCGTTTTATATACACCATGT | " | NA | NA | NA | NA | NA | NA |
| 306 | CcGM19810 | NA | AACGCTACCTGTACCACGCT | GCTATTTGCAAGGGGACAAA | " | NA | NA | NA | NA | NA | NA |
| 307 | CcGM19861 | P | CCGTGAGTATTGTCTTGAGCA | TTTAACGCATTATCTGGCCC | " | M | M | M | M | M | M |
| 308 | CcGM19876 | P | TATGTGAGCCCAGGGATAGC | TATGCCATCAAAACCCCAAT | " | M | M | P | P | P | P |
| 309 | CcGM19907 | P | CTTCCCGATCCAATGATACAA | TTTTGTGGCATGAAAGCAAG | " | P | M | M | M | M | M |
| 310 | CcGM19934 | P | AAATTTTTCCCTCCAACACAAA | AAGTGTACACCAAAACTAACTCAACA | " | M | M | M | M | M | M |
| 311 | CcGM20007 | P | TGTTATCAACTCAAAAACGCAA | TGTTATTTAACCTTTCACTATGCAACA | " | P | M | M | P | M | M |
| 312 | CcGM20042 | NA | ATTCGTGACTCATTTTGACTCTG | CCTATTAGTGAACAAGACTGACAAAG | " | NA | NA | NA | NA | NA | NA |
| 313 | CcGM20110 | P | TGGTCCATGTTCCTCACTCA | CCAATGAAAATGAGAACCTTCA | " | M | M | M | M | M | M |
| 314 | CcGM20114 | M | TTTCTATTGCTAAAAGTAATTGATTCG | TCTAATTTGGCCTAAACGGC | " | M | M | M | M | M | M |
| 315 | CcGM20115 | NA | TTTGGTTTGATTTGCTGCAC | CAAATGGAGGTCAAAACGGA | " | NA | NA | NA | NA | NA | NA |
| 316 | CcGM20155 | P | TTTCCTTTGATGCTTTTTCAA | TTTCTGCATGCCTTTCAACA | " | P | M | M | M | M | M |
| 317 | CcGM20163 | P | TCCACCAATCATTCGTCTCA | GGAGCGATATAAAGAAAAACACG | " | M | M | M | P | M | M |
| 318 | CcGM20190 | P | CCCCATGAATGGAGCAAATA | GAGAGAGTCCAACCTGGCAA | " | P | M | M | M | M | M |
| 319 | CcGM20208 | P | CCAACCTAAAACACAAACTCCTT | TTTCAACTCGTTACCCGACC | " | P | M | M | M | M | M |
| 320 | CcGM20296 | P | TTGGTGACGGCTTTCTAACA | TGCACATGCAAAGTGCCTAT | " | M | M | P | M | M | P |
| 321 | CcGM20307 | M | CCCTCAAAATCAAATAACGTCAA | AAGTTCAAGTCTCCCATTTGGT | " | M | M | M | M | M | M |
| 322 | CcGM20342 | P | TGTTGCAATGCTATCAACCTAAA | GGGATGTTACAATCCAACTTGA | " | M | M | M | M | M | P |
| 323 | CcGM20404 | P | TGGATCTCTCCACCTTTTCTCT | AATAGGTCGGCTTAAACGGG | " | M | M | M | M | M | M |
| 324 | CcGM20407 | P | TGGATCTCTCCACCTTTTCTCT | AATAGGTCGGCTTAAACGGG | " | M | P | M | M | M | P |
| 325 | CcGM20468 | NA | TAACTTTGTGGGGGCAGTTC | TGGTGTGTATGTCCCTTAAAGC | " | NA | NA | NA | NA | NA | NA |
| 326 | CcGM20469 | NA | TAACTTTGTGGGGGCAGTTC | GAAGGTTGATGATATAGACCTCCTTT | " | NA | NA | NA | NA | NA | NA |
| 327 | CcGM20486 | NA | CTCTAGTGCCACCAACCCAT | TTACACAATGGGTGGCAAAA | " | NA | NA | NA | NA | NA | NA |
| 328 | CcGM20504 | M | GGGCGATTATTCTTTTGGTTT | CAAGGATCCGTTTAGTTACGAAG | " | NA | M | NA | NA | NA | NA |
| 329 | CcGM20510 | M | GCCTTGCAACTGAGTCATCA | GTTCTTCTGCCATTCCCTCA | " | M | M | M | M | M | M |
| 330 | CcGM20512 | P | TGAGACACATGCCAAAACAT | CGGAACACTTTTAATTAGGCA | " | P | M | M | M | M | P |
| 331 | CcGM20569 | M | TCATTTGTCACCGCACAGAT | CTCCTCTTGCTCTTCGGTTG | " | NA | M | NA | NA | NA | NA |
| 332 | CcGM20577 | M | TAACTTTGTGGGGGCAGTTC | TCAGTTTTCCCATAACAAGTTAATCTC | " | NA | NA | M | M | NA | NA |
| 333 | CcGM20603 | P | AAAAGTCACGTCTCACAAGCA | AATTGTCTTTGAAAAGTGGATGA | " | P | M | M | M | M | M |
| 334 | CcGM20620 | P | CAACAACAAAAATTCAATTGTGAAA | CCCGTCATGTTATATCAATCAAA | " | M | M | M | M | M | P |
| 335 | CcGM20721 | P | TGATAAATTCTAAGTCCAACATGACAA | CGTTGCCATGCCTATTTTG | " | P | M | P | M | M | M |
| 336 | CcGM20775 | P | TTCATCAACTTCATTTTGGAGTG | TTCATACCAACCTCATCCCA | " | M | M | M | M | M | P |
| 337 | CcGM20942 | M | TGTCACAGCTTACATCTTTAAGTCC | TTTTAATAGCAATTTTTAGCACAAAGA | " | M | M | M | M | M | M |
| 338 | CcGM21015 | P | GTCACAGTGAATATTGTCCTGAGT | AATCCTAAACCAACCCGACC | " | M | M | M | M | M | M |
| 339 | CcGM21038 | P | GCAAACCCGAATTAACCTGA | CAATGAGTATTGTCTTGAGCAAATG | " | M | M | M | M | M | P |
| 340 | CcGM21044 | P | TTTTGAGACAACCATTTCAATGTA | AGGGTTGGGACTTTCCAAGA | " | M | M | M | M | M | M |
| 341 | CcGM21052 | M | CAACAATTATCCCCACACCC | GGACCCCAGAATGCCTATAA | " | NA | M | M | M | M | M |
| 342 | CcGM21056 | P | AGATAACCCTTTCTTTTATCATCATT | TTGAACCGACTCATTCAGTTTG | " | M | M | M | M | P | M |
| 343 | CcGM21072 | P | TGGACAAATTAACGATCAAAGA | TTTTCATTGAGACATGACTTTCA | " | M | M | M | M | M | M |
| 344 | CcGM21079 | P | TTTGGTTGGATTCGCAAAAT | GGTGTTGATTTTTATCCTTAAACCC | " | P | M | M | M | M | M |
| 345 | CcGM21170 | P | CCTAAAACCAAATTGAGTGCTTT | GCATGGTGTGCAGAAAGAGA | " | M | M | M | P | M | P |
| 346 | CcGM21174 | P | TACAAAGCAGAGAGCGCGTA | TTTTGTTTAATTAATGGATCAGGAG | " | M | M | P | M | M | P |
| 347 | CcGM21254 | M | ATCCCCGTCTCTATACCCGT | AGGGATGACAAATGTACCCG | " | M | M | M | M | M | M |
| 348 | CcGM21318 | M | GACCCTATATTGACCCTGAAAAA | GAAAATCTGTCTCTGCCGCT | " | M | M | M | M | M | M |
| 349 | CcGM21321 | P | AATTTGTTAAAACTCAACATGATTTC | TTTGCGCCATTTATTGCAT | " | M | M | M | P | M | M |
| 350 | CcGM21326 | M | GAGGGAGTGGGAGAGGAATC | TTTCTATGGTGGAAATTGCG | " | M | M | M | M | M | M |
| 351 | CcGM21332 | M | CCTTCCCCTTCACCAAATCT | CAAACCAGACATTCAAAATTCG | " | M | M | M | M | M | M |
| 352 | CcGM21344 | M | GGTGCTCAAGCACAAGCATA | GATCGAAGAGGTTGAGACGC | " | NA | M | NA | NA | M | NA |
| 353 | CcGM21347 | M | TTTCCAAAGTAAAGTCATCCTATGTT | GCCCCTTATTACTCGCATCA | " | M | M | M | M | M | M |
| 354 | CcGM21352 | M | CATGCATAGGAAGTAGCGGC | CGTCAATTTAAACACGGCCT | " | M | M | M | M | M | M |
| 355 | CcGM21476 | P | TTTAACCAACATCGACTACTACCA | TCGATTAAAGAAAAAGCCACAA | " | P | M | M | M | M | M |
| 356 | CcGM21502 | P | CCAACCTAAGACACAAAACTCG | CCACACCAATCTAATGAGCCT | " | M | M | M | M | M | M |
| 357 | CcGM21506 | P | GGTTGCTGGAGTGAAGTTCC | AAAGCCACGCCAAAGATTTA | " | P | M | M | P | M | M |
| 358 | CcGM21524 | M | TGAATCACCATTTTGTGTGGA | TTATCCCTAACCTAGGGCCG | " | M | M | M | M | M | M |
| 359 | CcGM21564 | M | GAAGGATGTTGATGTGGCCT | CGGTGGTTACTACCAAGGGA | " | M | M | M | M | M | M |
| 360 | CcGM21577 | M | CAAGAAAGCACAAACAATAAAGTGA | CGCATTCGTTTCAAGAACAA | " | M | M | M | M | M | M |
| 361 | CcGM21603 | M | GAAGGATGTTGATGTGGCCT | CGGTGGTTACTACCAAGGGA | " | NA | NA | M | NA | M | NA |
| 362 | CcGM21628 | P | CATCAATTACCATCCCCCAC | TGATTGGATTTCATGGGAACT | " | P | M | M | M | M | M |
| 363 | CcGM21634 | M | TGATTGGATTTCATGGGAACT | CATCAATTACCATCCCCCAC | " | M | M | M | M | M | M |
| 364 | CcGM21644 | P | TGATTGGATTTCATGGGAACT | CATCAATTACCATCCCCCAC | " | P | M | M | M | M | M |
| 365 | CcGM21651 | M | CAATGCTATAAACTTAAAACACGGAA | CTTTCCGAGTCGGGTAACAA | " | M | M | M | M | M | M |
| 366 | CcGM21693 | P | CATGGACGCGACACCTTAT | GGCCGATGATTTTTATGCAC | " | M | M | M | M | M | M |
| 367 | CcGM21706 | M | TCGTTATTATCGCCACTAGCAA | AACTCGTTTTTAGCGAGCCA | " | M | M | M | M | M | M |
| 368 | CcGM21715 | M | TCTACTTCTTACATTTTGAGACAATCA | AACAATGAGTGTGTGGTAGCAA | " | M | M | M | M | M | M |
| 369 | CcGM21732 | M | CATATCCATGGCAGTTGCTG | CTCCTGCCTGGAATCAAAAG | " | M | M | M | M | M | M |
| 370 | CcGM21761 | M | ATAACAAATGGGCCAAGTGC | CTTTTTATTTTGTTCTCTTGTAATGTG | " | M | M | M | M | M | M |
| 371 | CcGM21774 | P | TTTGACCTGTATCCCAACCC | TCTTGAGCAAGTGATCATTCAAA | " | P | M | M | M | M | M |
| 372 | CcGM21800 | M | AAGCATACATAGTTATCGAATCCA | TCTCCTTTGCGTCTTCTCAG | " | M | M | M | M | M | M |
| 373 | CcGM21816 | P | GGATCTCGTCCCAGATCAGA | ACCTGCAAAAGAGACTCCGA | " | M | M | M | M | M | M |
| 374 | CcGM21910 | P | TGAAATTTCTCGTGTGATTGTG | CCAAATCAGCTTGACTAACATTG | " | M | M | M | M | M | M |
| 375 | CcGM22072 | P | AAATGACCATCCAAACATTATTCT | TTCGACATGTGACTCGATCC | " | M | M | P | M | M | M |
| 376 | CcGM22109 | M | TTCACAATATCATCATGGTTAAGAGA | TGTGTGGATCATTTCCATGAAT | " | M | M | M | M | M | M |
| 377 | CcGM22116 | P | TGGTCAACAAAAATATAGAAATCAAGA | AGTGGGCAATAGTGTCAGGG | " | M | M | M | M | M | M |
| 378 | CcGM22134 | M | TGAAAATGAACATGAGGAAGAATG | TTTTAATACAAAAATTAACCAATGGC | " | M | M | M | M | M | M |
| 379 | CcGM22148 | M | TTGTTGAGACAAGAAGGCACA | GTGGAAGCAATTGCCAGAAT | " | M | M | M | M | M | M |
| 380 | CcGM22151 | P | AACACAAATGGAACATCGCA | ATCATCACCACTTCACCCGT | " | M | M | M | M | P | M |
| 381 | CcGM22222 | P | AAGATCAAATGAGAGGGGGC | CAAGGAATAATAAAACTTTATGCTCAA | " | M | M | M | M | M | M |
| 382 | CcGM22227 | P | ACAGATTTATTTGCCGCTGG | GGGATAAGATCACAGTCTTCTCG | " | P | M | M | M | M | M |
| 383 | CcGM22230 | M | GTCCAATTGTCTCGGATGCT | TTGTTCGAGCACCTCCTCTT | " | M | M | M | M | M | M |
| 384 | CcGM22341 | P | AACCCATTATCCGACCCAAT | TCAACCTAAAACACAAACCTCA | " | P | M | P | M | P | P |
| 385 | CcGM22410 | M | TTTCATGAAGGAGGTGAGAAA | GACCTAGGGCATCCCAATTT | " | M | M | M | M | M | M |
| 386 | CcGM22418 | P | CACTTGCCCTTTATTTGGTAGC | TGCATTTTCACTCCTCTCCA | " | M | M | M | M | M | M |
| 387 | CcGM22433 | M | TCAAATTAGGCCCAACAACA | CTAGGGCATCCCAATTTTCA | " | M | M | M | M | M | M |
| 388 | CcGM22436 | P | CCGATGCATCTCTTCTTAATTTT | CGACATACAATCCCAACAAGG | " | M | M | M | M | M | M |
| 389 | CcGM22440 | P | TTTTGATTTCTAGGAACCATATCAA | TGAATGAAGAAGTTTCAAACAACAA | " | P | M | M | M | M | M |
| 390 | CcGM22451 | M | GATGTTGTCACAATAGTTTATGCG | TCAACAAGGAGTTTGGAGCC | " | M | M | M | M | M | M |
| 391 | CcGM22458 | M | CAAGGCAGCGTCTACAATCA | ACATGCATGCTTTTGCCTTT | " | M | M | M | M | M | M |
| 392 | CcGM22543 | M | CATGTAATTTGACTTTTATTGTCGTTG | ACGATGTGTGGTCAAGTTGG | " | M | M | M | M | M | M |
| 393 | CcGM22559 | P | GCACTTCAAGGGGGTTTACA | GATCATTTGGACAGAAATATCAAA | " | M | M | M | M | M | P |
| 394 | CcGM22570 | P | CGTACGAATTCTCACATATTTTTCA | AATCGATTGAGGGTACTTGTGA | " | M | M | P | M | P | M |
| 395 | CcGM22598 | M | GTGCAGCTCAACAACACACC | AAATAATGTTTATCGGATGCTTG | " | M | M | M | M | M | M |
| 396 | CcGM22628 | M | TGAGAAAGGGCAACATGTCTT | TAGCACTTGCAACAGCCAAC | " | M | M | M | M | M | M |
| 397 | CcGM22684 | M | CGGCTGAAAATGATAATACAGTG | CCACATAGAAATAATCTCCCTTTTT | " | M | M | M | M | M | M |
| 398 | CcGM22728 | M | GCTCCCCAAAAGTCAATCAA | CACACCCAAACATGCTCACT | " | M | M | M | M | M | M |
| 399 | CcGM22747 | P | ACCTTTTTGCTCCGTTGAGA | ATGTCGAATTGACCTGAGGC | " | M | M | M | M | P | P |
| 400 | CcGM22805 | P | GGAAAACAGAGAGAGACAGAACAG | TCCCAATGCACTATGGCTA | " | M | M | P | M | P | P |
| 401 | CcGM22850 | P | CTCCCCTTTTGCAAACCATA | GAGGACATCGTATGCTTAACGTC | " | M | M | M | M | M | M |
| 402 | CcGM22867 | M | TATCCGCGAATAATACCCGA | AAAATGAGAATGGAGACGGG | " | M | M | M | M | M | M |
| 403 | CcGM22990 | P | AATTGAACTGTGAGTCCAAACA | GTGGAGCATCATCAGCCATA | " | P | P | M | P | M | M |
| 404 | CcGM22992 | P | TGAGTTTCAAATGATCCAGACC | GCACTCTACAACCTGCCACA | " | M | M | M | M | M | M |
| 405 | CcGM23005 | P | GCCTCAAACCCTTTTGTGAA | TATGAGTTGGGCAAAGGGAG | " | M | M | M | P | M | M |
| 406 | CcGM23006 | M | TCCATAACAAGGCAACGACA | ATCAAAGGTAGCGCACACG | " | M | M | M | M | M | M |
| 407 | CcGM23062 | P | TGGATTATAGAATATTTCAACCCATGT | CATTTTCAATGAATAACCAACAAAA | " | M | M | M | M | M | M |
| 408 | CcGM23078 | M | TTCCAAATGGCCATATTTATCA | TGGTCCAAGATTCCAAGGAG | " | M | M | M | M | M | M |
| 409 | CcGM23113 | M | TTTTGGGACTTCCATTTTTCA | GGCATGATAACGACCAATCC | " | M | M | M | M | M | M |
| 410 | CcGM23131 | P | GCGGTTTTGTAGCTCTGGAA | TGCAACCATACCACGACTGT | " | P | P | M | P | P | P |
| 411 | CcGM23147 | NA | TCACTTCCCTATATTTCTCCTAAAAA | CAATTGAAAAACCAATAAACATGA | " | NA | NA | NA | NA | NA | NA |
| 412 | CcGM23176 | P | CACGTGGCATCATCCTTATG | ATGTGTGCATGGTTGCATCT | " | M | P | M | P | P | M |
| 413 | CcGM23217 | M | CCTCACTTTGCCCATGTACC | TTCCTAAGCAACCACGTCCT | " | M | M | M | M | M | M |
| 414 | CcGM23262 | P | AACGGAGACCGTAGTGGTAA | TTGGTTTTCTTGAGCCTTCAG | " | P | M | P | P | P | M |
| 415 | CcGM23275 | M | CCAACCTGATCCAACCTGAT | TGACAATAAGTAATGTCCTAAGCAAAA | " | M | M | M | M | M | M |
| 416 | CcGM23316 | M | CCAACCTGATCCAACCTGAT | TGACAATAAGTAATGTCCTAAGCAAAA | " | M | M | M | M | M | M |
| 417 | CcGM23318 | M | GAGAGCGCGAATATCGTTTT | AGGAAAGGGCTTCCCATCTA | " | M | M | M | M | M | M |
| 418 | CcGM23321 | P | TGATATTGGTTTTCAGGGGC | TTCAGAAGCATCATCTTCCTTG | " | M | M | P | M | P | P |
| 419 | CcGM23354 | P | TACACGCATTTTTCCTCACG | TTGAGTCCCTACCAAGGAATG | " | M | M | M | M | M | P |
| 420 | CcGM23371 | P | GTGTTTTGAGCGATTGTGGA | GGAAAAGAACCCTAGACGCC | " | P | M | M | M | M | M |
| 421 | CcGM23387 | NA | CCCAGGATTTGTATTTGGGA | CAAGAGACCAACAAGGGCTC | " | NA | NA | NA | NA | NA | NA |

**^*^**Based on marker assay on eight pigeonpea genotypes
