# Supplementary material for: New Hypervariable SSR Markers for Diversity Analysis, Hybrid Purity Testing and Trait Mapping in Pigeonpea [Cajanus cajan (L.) Millspaugh]
Source: Front Plant Sci. 2017 Mar 31;8:377. doi: 10.3389/fpls.2017.00377 (PMC5374739; doi:10.3389/fpls.2017.00377)
Supplement: Supplementary file 4 [file Table4.doc]

**Supplementary Table 4**. SSR marker segregation analysis in the backcross population

| **S. No.** | **Marker Name** | **Number of individuals in backcross population** | | | **χ^2^-value** | ***p*-value** |
| --- | --- | --- | --- | --- | --- | --- |
|  |  | **ICPL 88039A type allele (P1)** | **Heterozygotes (H)** | **Missing** |  |  |
| 1 | ASSR0281 | 38 | 63 | 1 | 6.19 | 0.01 |
| 2 | ASSR1486 | 36 | 55 | 11 | 3.97 | 0.05 |
| 3 | CcGM01904 | 49 | 46 | 7 | 0.09 | 0.76 |
| 4 | CcGM03169 | 50 | 51 | 1 | 0.01 | 0.92 |
| 5 | CcGM03373 | 48 | 54 | 0 | 0.35 | 0.55 |
| 6 | CcGM07675 | 42 | 49 | 11 | 0.54 | 0.46 |
| 7 | CcGM08701 | 51 | 50 | 1 | 0.01 | 0.92 |
| 8 | CcGM08896 | 48 | 50 | 4 | 0.04 | 0.84 |
| 9 | CcGM09457 | 30 | 54 | 18 | 6.86 | 0.01 |
| 10 | CcGM10737 | 55 | 43 | 4 | 1.47 | 0.23 |
| 11 | CcGM11658 | 41 | 47 | 14 | 0.41 | 0.52 |
| 12 | CcGM12217 | 50 | 46 | 6 | 0.17 | 0.68 |
| 13 | CcGM12371 | 34 | 49 | 19 | 2.71 | 0.10 |
| 14 | CcGM12576 | 49 | 50 | 3 | 0.01 | 0.92 |
| 15 | CcGM12694 | 39 | 59 | 4 | 4.08 | 0.04 |
| 16 | CcGM14463 | 50 | 50 | 2 | 0 | 1.00 |
| 17 | CcGM14521 | 57 | 45 | 0 | 1.41 | 0.24 |
| 18 | CcGM15449 | 48 | 51 | 3 | 0.09 | 0.76 |
| 19 | CcGM16303 | 43 | 58 | 1 | 2.23 | 0.14 |
| 20 | CcGM16323 | 55 | 46 | 1 | 0.8 | 0.37 |
| 21 | CcGM16545 | 48 | 54 | 0 | 0.35 | 0.55 |
| 22 | CcGM16584 | 51 | 50 | 1 | 0.01 | 0.92 |
| 23 | CcGM16633 | 54 | 39 | 9 | 2.42 | 0.12 |
| 24 | CcGM16772 | 53 | 48 | 1 | 0.25 | 0.62 |
| 25 | CcGM17620 | 44 | 56 | 2 | 1.44 | 0.23 |
| 26 | CcGM17648 | 46 | 56 | 0 | 0.98 | 0.32 |
| 27 | CcGM18042 | 54 | 48 | 0 | 0.35 | 0.55 |
| 28 | CcGM18291 | 62 | 40 | 0 | 4.75 | 0.03 |
| 29 | CcGM18684 | 54 | 41 | 7 | 1.78 | 0.18 |
| 30 | CcGM19123 | 24 | 68 | 10 | 21.04 | 0.00 |
| 31 | CcGM19217 | 50 | 50 | 2 | 0 | 1.00 |
| 32 | CcGM19876 | 46 | 51 | 5 | 0.26 | 0.61 |
| 33 | CcGM20296 | 51 | 41 | 10 | 1.09 | 0.30 |
| 34 | CcGM22570 | 41 | 43 | 18 | 0.05 | 0.82 |
| 35 | CcGM23262 | 50 | 52 | 0 | 0.04 | 0.84 |
| 36 | CcGM23321 | 51 | 50 | 1 | 0.01 | 0.92 |
| 37 | CcM1841 | 49 | 47 | 6 | 0.04 | 0.84 |
| 38 | CcM2751 | 46 | 52 | 4 | 0.37 | 0.54 |
| 39 | CcM2855 | 37 | 60 | 5 | 5.45 | 0.02 |
| 40 | CZ681938 | 36 | 57 | 9 | 4.74 | 0.03 |
| 41 | HASSR002 | 56 | 44 | 2 | 1.44 | 0.23 |
| 42 | HASSR003 | 52 | 49 | 1 | 0.09 | 0.76 |
| 43 | HASSR005 | 50 | 48 | 4 | 0.04 | 0.84 |
| 44 | HASSR006 | 52 | 50 | 0 | 0.04 | 0.84 |
| 45 | HASSR022 | 43 | 45 | 14 | 0.05 | 0.82 |
| 46 | HASSR023 | 27 | 46 | 29 | 4.95 | 0.03 |
| 47 | HASSR041 | 51 | 49 | 2 | 0.04 | 0.84 |
| 48 | HASSR046 | 47 | 50 | 5 | 0.09 | 0.76 |
| 49 | HASSR057 | 50 | 49 | 3 | 0.01 | 0.92 |
| 50 | HASSR072 | 50 | 48 | 4 | 0.04 | 0.84 |
| 51 | HASSR081 | 34 | 60 | 8 | 7.19 | 0.01 |
| 52 | HASSR092 | 36 | 62 | 4 | 6.9 | 0.01 |
| 53 | HASSR099 | 47 | 46 | 9 | 0.01 | 0.92 |
| 54 | HASSR102 | 31 | 65 | 6 | 12.04 | 0.00 |
| 55 | HASSR107 | 40 | 54 | 8 | 2.09 | 0.15 |
| 56 | HASSR108 | 36 | 61 | 5 | 6.44 | 0.01 |
| 57 | HASSR110 | 47 | 44 | 11 | 0.1 | 0.75 |
| 58 | HASSR112 | 43 | 43 | 16 | 0 | 1.00 |
| 59 | HASSR115 | 42 | 56 | 4 | 2 | 0.16 |
| 60 | HASSR120 | 49 | 51 | 2 | 0.04 | 0.84 |
| 61 | HASSR128 | 49 | 52 | 1 | 0.09 | 0.76 |
| 62 | HASSR179 | 41 | 58 | 3 | 2.92 | 0.09 |
| 63 | HASSR189 | 46 | 56 | 0 | 0.98 | 0.32 |
| 64 | HASSR190 | 45 | 55 | 2 | 1 | 0.32 |
| 65 | HASSR194 | 46 | 56 | 0 | 0.98 | 0.32 |
| 66 | HASSR199 | 60 | 42 | 0 | 3.18 | 0.07 |
| 67 | HASSR201 | 48 | 54 | 0 | 0.35 | 0.55 |
| 68 | HASSR202 | 51 | 51 | 0 | 0 | 1.00 |
| 69 | HASSR205 | 50 | 51 | 1 | 0.01 | 0.92 |
| 70 | HASSR236 | 47 | 49 | 6 | 0.04 | 0.84 |
| 71 | HASSR277 | 34 | 61 | 7 | 7.67 | 0.01 |
| 72 | HASSR284 | 50 | 50 | 2 | 0 | 1.00 |
| 73 | HASSR292 | 61 | 26 | 15 | 14.08 | 0.00 |
| 74 | HASSR295 | 21 | 49 | 32 | 11.2 | 0.00 |
| 75 | HASSR300 | 27 | 59 | 16 | 11.91 | 0.00 |
