# Supplementary material for: New Hypervariable SSR Markers for Diversity Analysis, Hybrid Purity Testing and Trait Mapping in Pigeonpea [Cajanus cajan (L.) Millspaugh]
Source: Front Plant Sci. 2017 Mar 31;8:377. doi: 10.3389/fpls.2017.00377 (PMC5374739; doi:10.3389/fpls.2017.00377)
Supplement: Supplementary file 5 [file Presentation1.PPTX]

## Slide 1
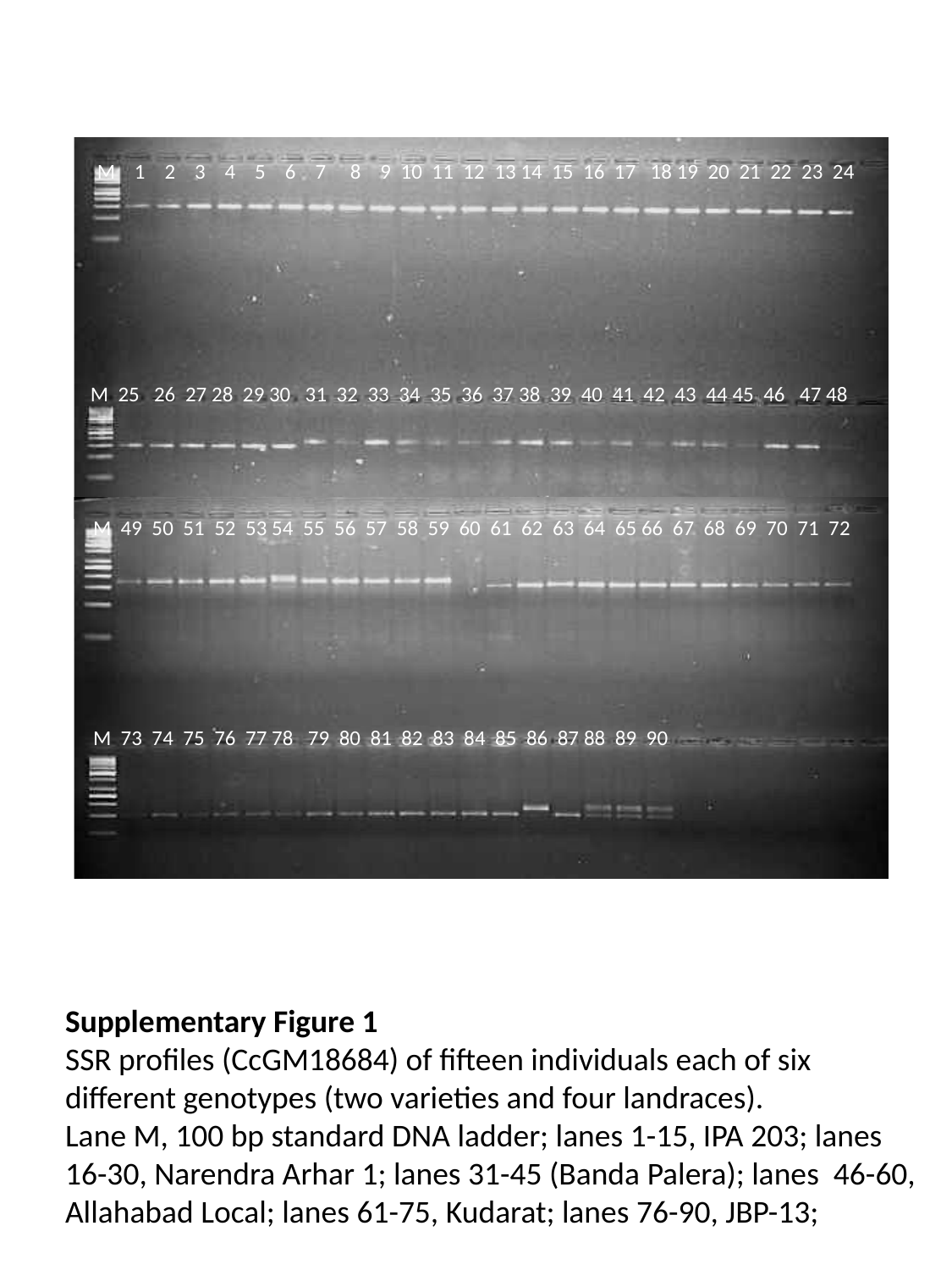

M 1 2 3 4 5 6 7 8 9 10 11 12 13 14 15 16 17 18 19 20 21 22 23 24
M 25 26 27 28 29 30 31 32 33 34 35 36 37 38 39 40 41 42 43 44 45 46 47 48
M 49 50 51 52 53 54 55 56 57 58 59 60 61 62 63 64 65 66 67 68 69 70 71 72
M 73 74 75 76 77 78 79 80 81 82 83 84 85 86 87 88 89 90
Supplementary Figure 1
SSR profiles (CcGM18684) of fifteen individuals each of six different genotypes (two varieties and four landraces).
Lane M, 100 bp standard DNA ladder; lanes 1-15, IPA 203; lanes 16-30, Narendra Arhar 1; lanes 31-45 (Banda Palera); lanes 46-60, Allahabad Local; lanes 61-75, Kudarat; lanes 76-90, JBP-13;
