# Supplementary material for: New Hypervariable SSR Markers for Diversity Analysis, Hybrid Purity Testing and Trait Mapping in Pigeonpea [Cajanus cajan (L.) Millspaugh]
Source: Front Plant Sci. 2017 Mar 31;8:377. doi: 10.3389/fpls.2017.00377 (PMC5374739; doi:10.3389/fpls.2017.00377)
Supplement: Supplementary file 6 [file Presentation2.PPT]

## Slide 1
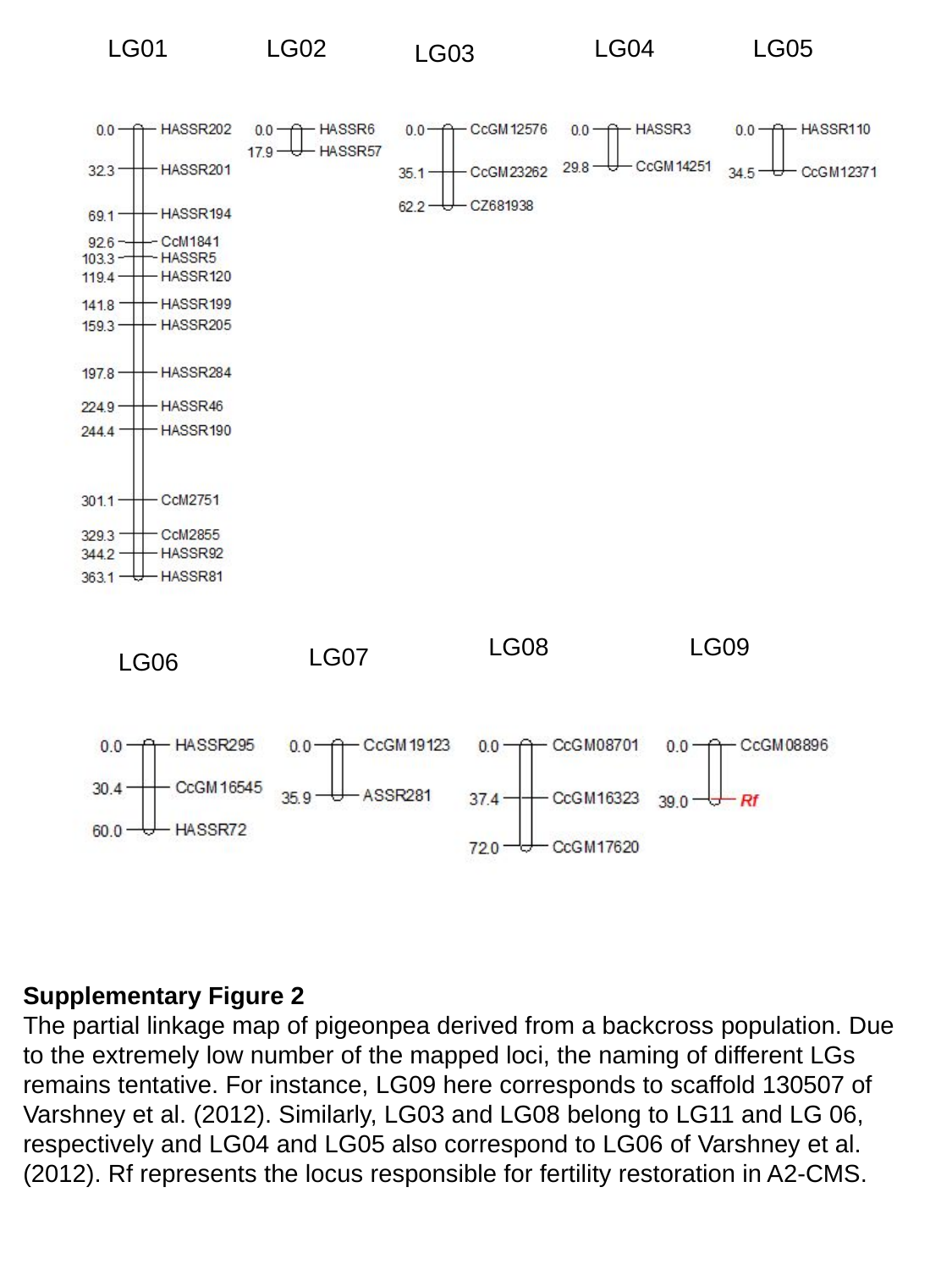

LG01
LG02
LG04
LG05
LG03
LG08
LG09
LG07
LG06
Supplementary Figure 2
The partial linkage map of pigeonpea derived from a backcross population. Due to the extremely low number of the mapped loci, the naming of different LGs remains tentative. For instance, LG09 here corresponds to scaffold 130507 of Varshney et al. (2012). Similarly, LG03 and LG08 belong to LG11 and LG 06, respectively and LG04 and LG05 also correspond to LG06 of Varshney et al. (2012). Rf represents the locus responsible for fertility restoration in A2-CMS.
